# Machine Learning for Predicting 12-Month Survival after Liver Transplantation: Temporal Validation, Calibration, and Decision-Analytic Evaluation

**DOI:** 10.34133/csbj.0091

**Published:** 2026-06-05

**Authors:** Jose Ricardo de Oliveira, Adriano Galindo Leal, Rodrigo Luiz Macacari, Ben-Hur Ferraz-Neto

**Affiliations:** ^1^ Artificial Intelligence and Analytics Department, Institute for Technological Research, São Paulo 05508-901, SP, Brazil.; ^2^ Hospital das Clinicas (HCFMUSP), Faculdade de Medicina, Universidade de São Paulo, São Paulo 05403-010, SP, Brazil.; ^3^ Instituto do Figado, Rede Américas, São Paulo 04534-011, SP, Brazil.

## Abstract

Accurate prediction of posttransplant survival is critical for optimizing liver allocation under organ scarcity. Although the Model for End-Stage Liver Disease (MELD) is widely used for prioritization, it was not designed to estimate posttransplant outcomes, limiting its utility for individualized risk stratification. We developed and temporally validated machine-learning models to predict 12-month survival after liver transplantation using routinely collected donor–recipient variables from a real-world cohort. Sequential temporal validation was used to approximate prospective deployment. Performance was evaluated in terms of discrimination (receiver operating characteristic–area under the curve [ROC-AUC] and precision–recall area under the curve), calibration (Brier score), and decision-analytic evaluation using decision curve analysis. Class imbalance was addressed through cost-sensitive learning, and predicted probabilities were recalibrated with Platt scaling. Across temporal validation windows, discrimination remained modest, with ROC-AUC values below 0.70 and reaching 0.599 in the final window, reflecting the complexity of posttransplant outcomes in heterogeneous clinical populations. Calibration remained relatively stable (Brier score ≈ 0.18). Within the evaluated threshold range, the machine-learning models yielded a positive net benefit and generally exceeded the transformed MELD baseline in decision-analytic comparisons. However, these findings should be interpreted cautiously given the modest discrimination and the exploratory nature of the MELD transformation used for cross-model comparison. Logistic regression provided the most consistent balance of discrimination, calibration, and net benefit. Overall, calibrated models with modest discrimination may still offer complementary risk information. However, further external validation, threshold-specific evaluation, and prospective testing are required before such models can be considered for operational decision support.

## Graphical Abstract

**Figure:**
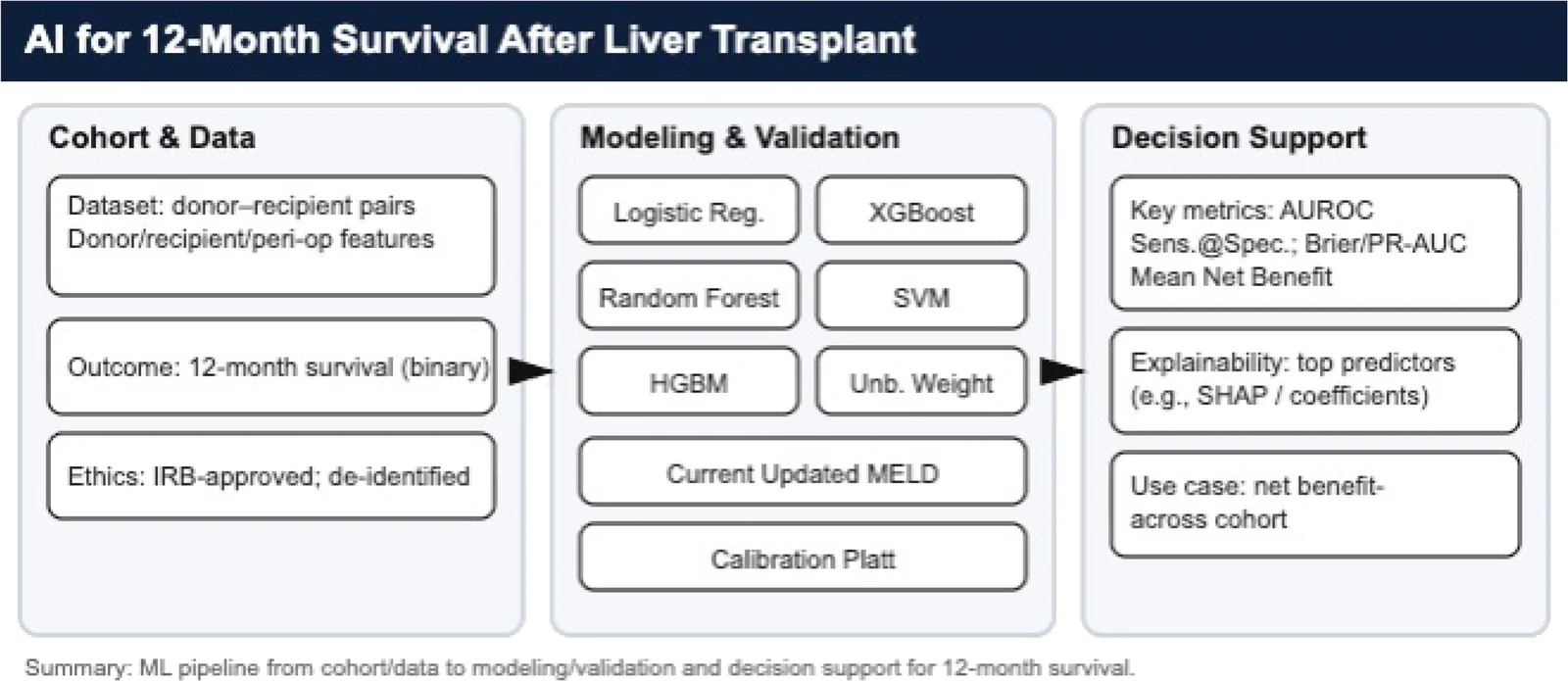

## Introduction

In the *Song of the Cell*, Mukherjee [1] defines the central systems responsible for maintaining the body’s homeostasis: “The liver, pancreas, brain, and kidney are the four main organs of homeostasis. (...) The liver, among many other functions, prevents us from being flooded with toxic products such as ethanol.” This statement emphasizes the liver’s crucial role in metabolism and detoxification, which are vital to the body’s internal regulation.

Liver problems can lead to self-poisoning, making the proper functioning of this organ vital for everyone, even if a transplant is necessary. According to de Luna Nascimento et al. [2], Brazil was the third country in the world in the number of liver transplants, with 2,033 procedures performed in 2020. However, this number is far from cause for celebration, as in the same period, the transplant waiting list had 5,254 patients.

Liver transplantation is a widely accepted treatment for patients with severe liver disease [3]. However, the limited availability of donor organs remains a major constraint, and the resulting imbalance contributes to substantial mortality among patients on the waiting list.

The medical and academic community has made many efforts to improve organ allocation criteria and ensure that the most severe cases have priority access to liver transplants. One of the most substantial advances in this context is the use of the Model for End-stage Liver Disease (MELD) [4], a widely accepted statistical model to classify the risk of short-term mortality in patients without transplantation, as noted by de Luna Nascimento et al. [2]. Moreover, Wingfield et al. [5] emphasize that the MELD method is considered the gold standard for prioritizing patients on the waiting list, helping to make fairer and more effective decisions in organ distribution.

However, graft acceptance by medical teams, combined with recipient prioritization and organ acquisition, creates a highly complex and challenging scenario to model.

According to Cruz-Ramírez et al. [3], the decision to accept a graft is a subjective problem influenced by dozens or even hundreds of variables. This process involves clinical, logistical, and ethical factors, making careful analysis essential to minimize errors and ensure that the available organ is allocated to the patient with the greatest need and the best prognosis. Another critical factor is the need to avoid wasting grafts that go unused due to multiple rejections by medical teams, which can cause ischemia time to exceed surgical limits. Decision-support tools that inform recipient-specific graft acceptance are potentially relevant to improving transplant assessment and could support posttransplant risk stratification and more efficient use of available organs. Research gap and study objective: Despite advances in predictive modeling, most studies in liver transplantation have focused primarily on discrimination metrics, often overlooking calibration and clinical utility, both of which are relevant to real-world decision-making. The predominant reliance on receiver operating characteristic–area under the curve (ROC-AUC) as the primary performance metric also limits the assessment of model behavior in imbalanced, high-stakes settings such as liver transplantation. Collectively, these limitations highlight a critical gap: the lack of comprehensive evaluation frameworks that integrate temporal validation, calibration assessment, and decision-analytic analysis. To address these limitations, this study aims to develop and rigorously validate machine-learning models to predict 12-month posttransplant survival in a real-world, time-dependent deployment framework. In addition to discrimination, this study emphasizes probability calibration and decision-analytic evaluation so that model outputs can be examined not only in terms of ranking performance but also in terms of their potential usefulness as complementary clinical information. Contributions: This study makes 3 main contributions. First, it implements a temporally structured validation strategy that mimics real-world model deployment, providing a more realistic estimate of model performance over time. Second, it adopts a comprehensive evaluation framework that extends beyond discrimination metrics by incorporating calibration and decision curve analysis (DCA), thereby broadening the assessment of model behavior. Third, it performs an exploratory comparison against MELD-based strategies to examine whether multivariable machine-learning models provide incremental decision-analytic information beyond traditional severity scores.

## Literature Review

### AI on liver transplantation and decision-making concerns

According to Cruz-Ramírez et al. [3], risk classification methods for recipient mortality, such as MELD, use logistic regression to classify patients. However, this technique can neither capture graft failure or posttransplant mortality nor predict whether the recipient will survive or for how long after transplantation.

The decision to accept or reject a graft is still made indirectly and subjectively by the medical team, taking into account the clinical history, blood tests, steatosis measurements, and other factors, as noted by Cesaretti et al. [6]. These authors note that the primary assessment is performed by medical teams based on the graft’s texture and color, achieving a precision of 86%. Although this is a satisfactory rate, the analysis remains subjective. Lee et al. [7] report that beyond the subjective factor, organ donors have become older, with more comorbidities, and donation criteria have expanded to include organs of donors with postmortem blood circulation problems. The donor–recipient matching process is becoming increasingly critical as transplant waiting lists grow and variations in the acceptance process increase the risk that grafts are unused (late withdrawals, increased ischemia time). Although neural network methods show considerable promise for augmenting MELD-based allocation [3], graft acceptance by transplant teams remains a pivotal constraint that complicates allocation decisions, reflecting subjective clinical considerations not fully captured by current predictive models. Evidence from Cesaretti et al. [6] indicates that convolutional neural networks can estimate liver graft steatosis, enabling more rigorous pretransplant viability assessment and, when integrated into clinical decision-making, more precise graft allocation, higher transplant success rates, and shorter patient waiting times. According to Kwong and Asrani [8], the viability time of a donated organ is a key factor in predicting posttransplant recovery and maximizing the patient’s survival rate. The authors note that artificial intelligence (AI) has been at the forefront of graft acceptance and can be used to estimate the recipient’s probability of survival in conjunction with the MELD score, thereby assisting medical teams.

However, according to Golse [9], AI should always serve as a support tool, and medical teams should always make the final decision regarding graft acceptance. Predicting whether a specific graft can be used for a patient is crucial for ensuring recipient survival, expediting the priority-list process, and reducing organ waste.

According to Drezga-Kleiminger et al. [10], the allocation of a graft between a donor and a recipient is a highly complex task that involves intricate predictions based on various donor and recipient variables. However, these authors also state that AI, when used in other medical fields, can be as accurate as, or even more accurate than, medical professionals.

More broadly, recent studies published in the *Computational and Structural Biotechnology Journal* support integrating AI into complementary stages of transplant-related and liver-focused clinical workflows.

In transplantation, Fayzullin et al. [11] demonstrated that AI-assisted digital pathology can reduce subjectivity and improve the reproducibility of kidney allograft rejection assessment by quantitatively analyzing prognostic morphological patterns in biopsy images. In the liver field, Long et al. [12] introduced LiverSCA, a user-friendly single-cell atlas for hepatocellular carcinoma that supports mechanistic exploration of cell populations, differential expression, cellular cross talk, and trajectory inference. Although these studies do not directly address donor–recipient survival prediction, they illustrate a convergent trend toward computationally assisted, data-rich, and clinically interpretable decision support in hepatology and transplantation, which aligns with the rationale of the present study.

### MELD as state of the art and its issues

The MELD score is the most widely accepted statistical model for classifying the short-term risk of death in patients without a transplant (3-month risk) [2]. Since the MELD model was introduced in 2000 by Malinchoc et al. [4], several countries worldwide, including Brazil, have adopted it, successfully prioritizing the most urgent cases.

Based on standard, auditable clinical tests, the method can be established within a clear policy that is open to all members of society, creating a priority list based on classification rather than on subjective or spurious criteria. MELD aims to prioritize transplantation for patients with the most urgent need.

As mentioned by Vasconcellos and Zamith [13], the MELD score is calculated as follows:

$$\begin{array}{c} \begin{array}{ll} \text{MELD}\; & =10\left[0.957\;\ln\left(\text{serum creatinine}\right)\right.\\ & +0.378\;\ln\left(\text{total bilirubin}\right)\\ & +1.120\;\ln\left(\mathrm{INR}\right)\\ & \left.+0.643\;\right] \end{array} \end{array}$$ (1)

MELD−Na=MELD+1.32137−serumsodium−0.033MELD137−serumsodium (2)

MELD3.0=1.33Ifemale+4.56lntotalbilirubin+0.82137−serumsodium−0.24137−serumsodiumlntotal bilirubin+9.09lnINR+11.14lnserum creatinine+1.853.5−serumalbumin−1.833.5−serumalbuminlnserum creatinine+6 (3)

Notes: Units: Serum creatinine and total bilirubin are in milligrams/deciliter, serum albumin is in grams/deciliter, serum sodium is in millimoles/liter, and INR (international normalized ratio) is dimensionless. $\ln\left(\cdot \right)$ is the natural logarithm. Before applying logarithmic transformations, lower-bound truncation was applied to avoid values below 1.0: serum creatinine, total bilirubin, and INR values <1.0 were replaced with 1.0. For MELD/MELD-Na, candidates with ≥2 dialysis sessions in the prior 7 d were assigned serum creatinine = 4.0 mg/dl. For MELD 3.0, the following additional policy truncations were applied before score calculation: creatinine was restricted to the interval [1.0, 3.0] mg/dl, albumin to [1.5, 3.5] g/dl, and sodium to [125, 137] mmol/l. Scores were rounded to the nearest integer for reporting.

However, although MELD measures the probability of death in 3 months for patients without a transplant, it does not provide information on the patient’s expected survival after transplantation.

### Survival of patients after liver transplantation

An article by Coelho et al. [14] discusses the advances in clinical care and surgical techniques that have contributed to improved survival rates among patients undergoing liver transplantation. In addition, according to Coelho et al. [14], the first posttransplant year is a particularly critical period for recipient survival, with a substantial proportion of deaths occurring during this interval. However, most deaths occur within this period, particularly during the first 3 months, and are often associated with primary graft dysfunction, technical complications, and infections.

Improving the allocation of donated grafts to recipients would not only reduce 12-month mortality rates but also increase recipient survival and improve graft utilization. Death within 12 months also represents a loss of realized graft benefit and reduces the overall efficiency of graft utilization.

**Fig. 1.**
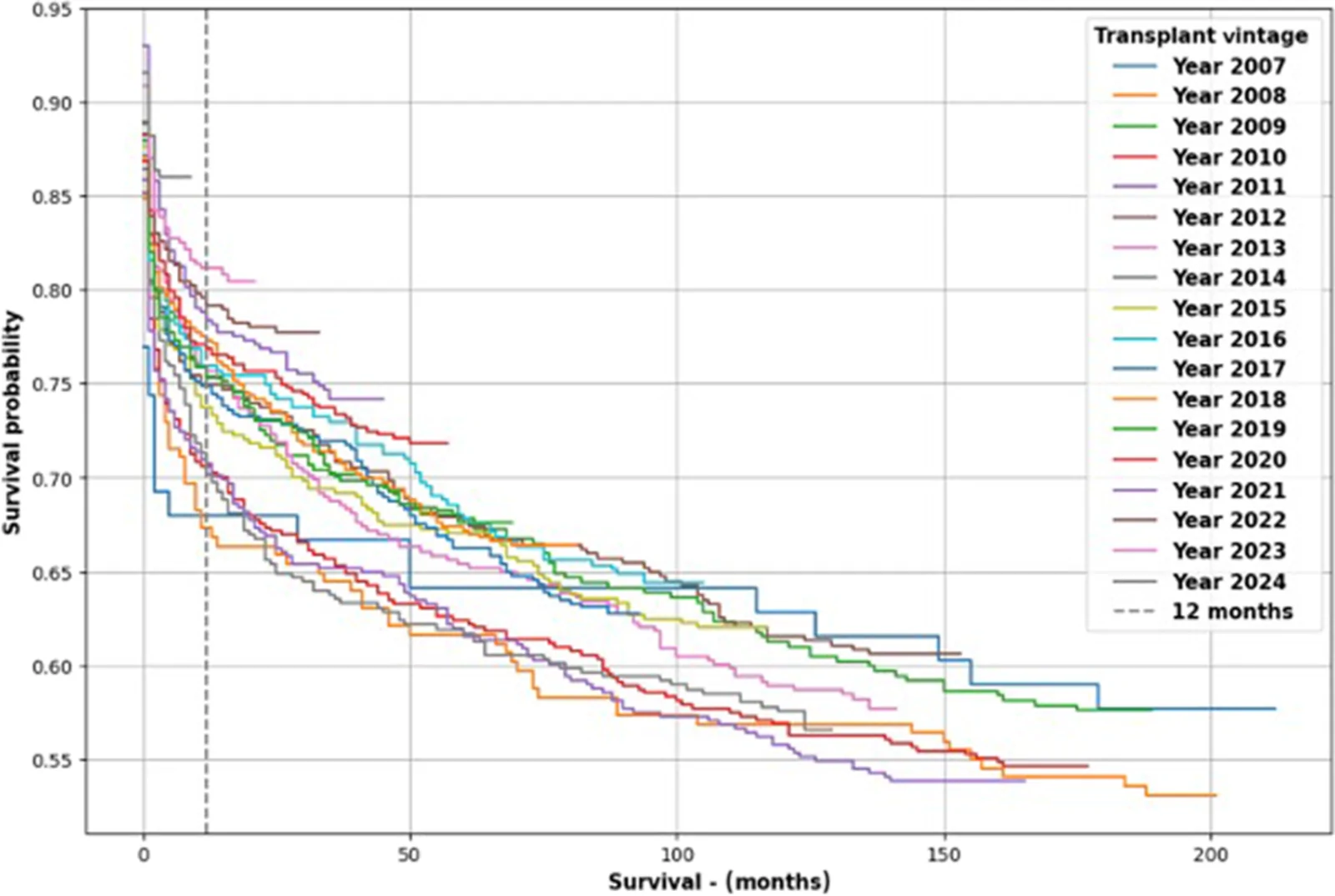
Kaplan–Meier survival curve for liver transplantation in the State of São Paulo, Brazil, by year.

According to data from the São Paulo State Health Secretariat, the Kaplan–Meier survival analysis of liver transplant recipients, as shown in Fig. 1, indicates that the first 12 months are the most critical for posttransplant survival. Currently, between 70% and 80% of the recipients survive beyond this period.

### Main algorithm applications of AI in graft allocation for transplant recipients

Applying AI algorithms to predict whether a donor is compatible with a recipient involves a complex issue: class imbalance. Traditional statistical methods are effective at predicting the majority class (the recipient survives the transplant) but tend to perform poorly for the minority class (patient death or graft rejection), as noted by Calleja Lozano et al. [15]. To address this issue, the authors propose using neural networks and random forests, as both techniques are well known for effectively handling imbalanced data. In this study, both neural networks and random forests outperformed the MELD score in predicting 3-month survival on the dataset used.

In an article by Guijo-Rubio et al. [16], the authors expanded the range of algorithms used and compared them not with MELD but with other statistical models such as Survival Outcomes Following Liver Transplantation (SOFT) and Balance of Risk (BAR) scores. In addition to neural networks and random forests, they applied gradient boosting, support vector machine (SVM), and logistic regression. All AI-based methods outperformed statistical models. An interesting point raised by the authors is that the algorithms were designed to predict survival over various time horizons: 3 months, 1 year, 2 years, and 5 years.

In the study by Raju and Sathyalakshmi [17], the authors introduced transformers and used the FT-Transformer algorithm to predict survival over a specified time frame. The transformer demonstrated performance comparable to that of neural networks and random forests.

Lau et al. [18] conducted a study at Austin Health (Australia) to compare the donor risk index with AI algorithms, such as artificial neural network and random forest, obtaining better results with the latter than with donor risk index.

Table 1 presents a summary of the ROC-AUC metric of the studies mentioned above, with respect to liver transplant survival at 3 and 12 months, considering the AI algorithms most frequently cited.

**Table 1.** Liver graft survival ROC-AUC

Study	AI model	No. of reg.	3-month	12-month
Spanish multicentric study [15]	ANN	1,003	0.80	NR
King’s College Hospital [15]	ANN	858	0.94	0.78
UNOS [16]	ANN	39,189	0.54	0.56
UNOS [16]	LR	39,189	0.63	0.63
UNOS [16]	RF	39,189	0.62	0.61
Austin Health [18]	ANN	90	0.72	NR
Austin Health [18]	RF	90	0.56	NR
UNOS [17]	RF	NR	0.92	NR
UNOS [17]	ANN	NR	0.83	NR
UNOS [17]	LR	NR	0.84	NR

NR, not reported in the original study; ROC-AUC, receiver operating characteristic–area under the curve; AI, artificial intelligence; ANN, artificial neural network; UNOS, United Network for Organ Sharing; LR, logistic regression; RF, random forest

Regarding AI algorithms, the following have been used in various studies to predict post-liver transplantation survival: neural networks, random forest, logistic regression, gradient boosting, SVM, and transformer (FT-Transformer), with the first 3 being the most cited.

## Data Extraction and Preparation

### Dataset description

Regarding the construction of 12-month survival predictive models after a liver transplant, the São Paulo State Health Secretariat provided, in tabular format (Excel), all information from the transplant registry up to the extraction date (2024 October 31). It is worth noting that the dataset included both the transplants performed and all recipients on the waiting list who had not yet undergone transplantation. To protect the identities of each patient (donor and recipient) and to comply with Brazil’s General Data Protection Law, all data were anonymized.

Analyzing the transplant cohort as a whole (Appendix F), the mean posttransplant survival was 64.66 months (5 years and 4 months), with a standard deviation of 60.24 months. The outcome distribution was imbalanced, with 26.71% mortality within 12 months. This imbalance was considered in model development and evaluation to avoid biased performance estimates.

This study originated from a master’s dissertation [19] and was conducted using fully anonymized, retrospectively collected registry data. Informed consent was waived because individual identification was not possible, and the dataset was provided and authorized for research and scientific publication by the São Paulo State Health Secretariat, in compliance with Brazil’s General Data Protection Law (Lei Geral de Proteção de Dados Pessoais). Code and configuration are available through a controlled-access Zenodo archive at https://zenodo.org/records/18213728 [20]; environment specifications and exact commands for full replication are provided. All experiments were conducted using fixed random seeds to ensure reproducibility.

#### Data extraction and cohort construction

From the São Paulo State transplant registry, we identified 9,603 liver transplants. After restricting the study window to complete digital records, 9,408 cases remained (transplants after 2007). Dual-organ transplants (*n* = 263), recipients <18 years (*n* = 466), lost to follow-up within 12 months (*n* = 293), and graft loss at time 0 (*n* = 8) were excluded, yielding 8,378 cases. After removing rows with missing key predictors (creatinine, total bilirubin, INR, albumin, sodium, etc.) or inconsistent timestamps, 7,902 records were available. To ensure a full 12-month observation window, we excluded cases after the data freeze (i.e., 2023 to 2024), yielding 7,098 donor–recipient pairs for model development and evaluation. Cohort selection followed a structured filtering process based on clinical and data-quality criteria, as summarized in Table 2.

**Table 2.** Cohort construction and exclusions (12-month survival analysis)

Step	*N*
All liver transplants in registry	9,603
Digital records within study window	9,408
Exclude dual-organ transplants	−263
Exclude recipients <18 years	−466
Exclude lost to follow-up within 12 months	−293
Exclude graft loss at time 0	−8
Available after clinical exclusions	8,378
Remove missing key predictors/timestamp issues	−476
Available after data-quality filters	7,902
Exclude index dates without ≥12 months of potential follow-up (2023–2024)	−804
Final analysis cohort	7,098

Missingness was predominantly observed across variables and was associated with donor, recipient, and data collection practices. However, multiple imputation is commonly applied; it could introduce model-dependent assumptions and potentially distort clinically meaningful relationships. Therefore, a complete-case analysis was adopted as a conservative approach to preserve data integrity and avoid introducing synthetic values into key prognostic variables.

The potential impact of this decision on selection bias is acknowledged and discussed in the “Limitations” section.

The original registry contained 126 variables. Variables corresponding to administrative identifiers, duplicate information, and attributes unrelated to liver transplant were excluded during preprocessing, resulting in 77 attributes related to donor characteristics, recipient clinical variables, and transplant logistics.

To reduce redundancy and mitigate multicollinearity among highly correlated severity scores, only the current updated MELD was retained. This variable reflects the most recent and clinically relevant assessment of liver disease severity before transplantation, incorporating both laboratory values and diagnostic adjustments.

Other MELD-related variables (MELD at listing, updated MELD, and current MELD based solely on laboratory values) were excluded because they represent earlier or less comprehensive clinical states and provide overlapping information.

The primary outcome was 12-month posttransplant survival, defined as a binary variable indicating whether the recipient was alive (positive class) at 12 months posttransplant. Patients who died within this period were classified as the positive class for mortality analyses in the “Minority-class (mortality) performance analysis” section.

#### Exploratory univariate analysis

Baseline characteristics, stratified by 12-month survival, are presented in Appendix B. Several clinically relevant variables differed significantly between patients with survival ≥12 months and those who died within 12 months. Variables reflecting disease severity and allocation status, such as current updated MELD, prioritization status, and diagnosis, showed the most pronounced differences (P<0.001).

Recipient-related factors, including age and sex, were also significantly associated with the outcome. Additionally, selected donor-related biochemical markers demonstrated moderate differences between groups. However, several physiological donor variables, such as creatinine and sodium, did not show statistically significant differences.

Given the large sample size, statistically significant differences should be interpreted with caution, as even small effect sizes may yield low *P* values. Therefore, clinical relevance and multivariable model performance should be considered alongside univariate findings.

Among the 77 attributes evaluated, 24 variables showed statistically significant differences (*P* < 0.05) between the 2 outcome groups. They were therefore considered potentially relevant for distinguishing patients who survived beyond 12 months from those who did not.

No correction for multiple comparisons was applied, as this analysis was intended for exploratory and descriptive purposes only. Therefore, *P* values should be interpreted with caution and not as confirmatory evidence.

### UMAP dataset representation

Dimensionality reduction techniques were used to visualize the high-dimensional transplant dataset. Uniform manifold approximation and projection (UMAP) was selected due to its ability to capture nonlinear structures while preserving local neighborhood relationships [21]. For comparison, principal component analysis (PCA), a linear method, was also applied.

**Fig. 2.**
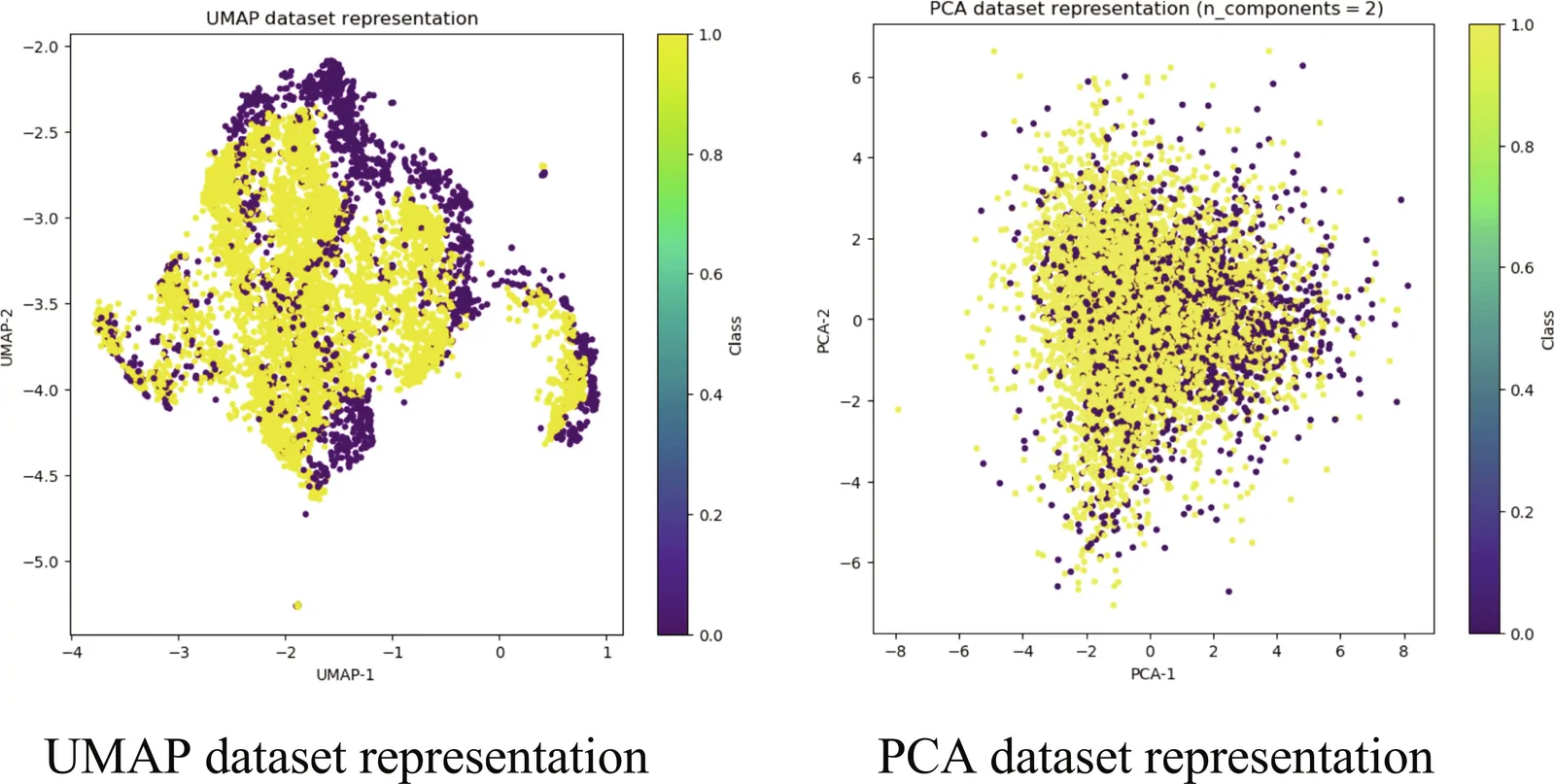
Low-dimensional representations of the dataset using uniform manifold approximation and projection (UMAP) and principal component analysis (PCA).

The 2-dimensional UMAP embedding (Fig. 2A) reveals nonlinear structures in the dataset; however, class separation remains limited. Although certain regions show relative enrichment of one outcome, a substantial degree of overlap persists, indicating that the classes are not trivially separable in an unsupervised representation.

As the embedding was generated without supervision (target weight = 0), the observed structure reflects intrinsic relationships in the data rather than label-driven separation. Additionally, density patterns are influenced by class imbalance, with the majority class dominating the embedding.

In contrast, the PCA projection (Fig. 2B) shows a largely homogeneous and overlapping distribution with an approximately elliptical structure, suggesting that the main axes of variance are not aligned with outcome-related differences. A *k*-nearest neighbors classifier (*k* = 10) applied to the PCA-reduced space achieved an accuracy of 0.70, further supporting the limited discriminative capacity of linear projections.

Overall, both PCA and UMAP indicate that posttransplant survival is not linearly separable, reinforcing the need for models capable of capturing complex nonlinear interactions among clinical variables.

The interpretation of the UMAP hyperparameters is provided in Appendix D.

## Model Development and Evaluation

### Models selected and hyperparameters

The objective of predictive models, as identified in the literature, is to determine the likelihood of the survival of the donor–recipient pair within 12 months. For this purpose, the positive class represents pairs that survive during this time frame, whereas the negative class denotes those that do not.

Based on previous research, the following supervised learning models were selected and trained: random forest, SVM, logistic regression, XGBoost, and gradient boosting. The full set of hyperparameters and their search spaces are detailed in Appendix C.

Deep learning architectures (e.g., artificial neural networks, TabNet, and autoencoders) were explored in preliminary experiments but excluded from the final analysis due to inferior performance and reduced calibration stability compared with those of classical machine-learning models. In contrast, classical machine-learning approaches, particularly logistic regression and tree-based ensemble models, demonstrated more stable performance and better calibration. Given the objective of developing reliable and interpretable decision-support tools for clinical use, the final analysis focused on models that best balanced predictive performance, robustness, and interpretability.

Model selection during hyperparameter optimization was based on precision–recall area under the curve (PR-AUC), Brier score, and mean net benefit, computed on the validation folds.

### Sliding window and time series split for training

To ensure a realistic evaluation of model performance while preserving the dataset’s temporal structure, we adopted a sliding-window temporal validation strategy combined with time-aware cross-validation for hyperparameter optimization.

Specifically, models were trained using a rolling training window of 4 consecutive years, and performance was evaluated in the subsequent year. This design mimics real-world clinical deployment, where models trained on historical data are applied to future transplant candidates. For example, models trained on data from 2007 to 2010 were evaluated in 2011, after which the training window was moved forward by 1 year (e.g., 2008 to 2011 → test in 2012). This process was repeated sequentially until the end of the study period.

Within each training window, hyperparameter optimization was performed using a time-aware cross-validation procedure based on TimeSeriesSplit with 3 folds. Unlike conventional *k*-fold cross-validation, which randomly partitions the dataset, TimeSeriesSplit preserves the chronological order of observations and ensures that validation data always follow the training data. This design prevents information leakage from future observations, which is a critical consideration when working with longitudinal clinical datasets such as transplant registries.

In each training window, the available data were therefore partitioned into 3 sequential folds, with earlier observations used to train the model and subsequent observations serving as validation sets. Hyperparameter optimization was performed using RandomizedSearchCV, sampling configurations from predefined distributions, and selecting the best-performing model based on validation performance.

This combined strategy of sliding temporal windows and time-aware cross-validation provides a robust and clinically realistic framework for evaluating predictive models in transplant outcome prediction, ensuring that performance estimates reflect the practical scenario of predicting outcomes for future transplant recipients using only historical data.

This procedure yielded 12 temporal validation windows, with the final window (train: 2018 to 2021; test: 2022) used for the primary model comparison.

### Imbalance handling

To address the class imbalance, a cost-sensitive learning strategy was incorporated during model training. The dataset exhibits a moderate class imbalance, with approximately 27% of the cases corresponding to mortality within 12 months after transplantation. Instead of applying resampling techniques, we adopted a class-weighting approach that assigns greater importance to the minority class during model optimization.

Specifically, balanced class weights were applied during training to ensure that each observation’s contribution to the loss function is inversely proportional to its class frequency. In practice, this was implemented by computing sample weights using the class weight = “balanced” strategy, which adjusts the effective weight of each sample based on the relative frequency of the outcome classes in the training data.

Importantly, this implementation corresponds to an explicit form of cost-sensitive learning, in which misclassification costs are directly incorporated into the training process through observation-level weights. This strategy addresses class imbalance without modifying the original data distribution, thus avoiding potential biases introduced by oversampling or synthetic data generation techniques.

This approach was chosen to improve sensitivity to the minority class while preserving the original data distribution, particularly in the context of threshold-dependent metrics and clinical decision analysis.

### Model training and calibration

After model training, an additional probability calibration step was performed to improve the reliability of predicted probabilities. Calibration is particularly important in clinical decision-support systems, where predicted probabilities are often interpreted as estimates of patient risk and can directly influence medical decision-making.

To address this, we applied post hoc probability calibration using the Platt scaling method, implemented through the CalibratedClassifierCV framework with the sigmoid calibration function. This method fits a logistic regression model that maps the raw model outputs to calibrated probabilities, correcting systematic deviations between predicted probabilities and observed outcome frequencies.

The calibration procedure was performed using 5-fold cross-validation, ensuring that the calibration model was trained and validated on separate subsets of the training data. This approach reduces the risk of overfitting during calibration while providing a more reliable mapping from predicted scores to actual event probabilities.

The calibrated probabilities were subsequently used for all analyses presented during this study. By improving the alignment between predicted probabilities and observed outcomes, this calibration step enhances the clinical interpretability and reliability of the model output.

A complete summary of model performance metrics across all temporal validation and test windows is presented in Tables A1 to A7 in Appendix A.

### Model performance in the final temporal window (2018 to 2021 → 2022)

To train predictive models to estimate the probability of survival at 12 months posttransplant, data from the years 2023 and 2024 were deliberately excluded. This decision was made to avoid introducing bias due to incomplete outcome data, as some transplants from these years may not have reached the full 12-month follow-up period. Consequently, the final training dataset comprised 7,098 donor–recipient pairs.

Operating thresholds (Youden’s J) were selected on the validation set and applied unchanged to the held-out test set. Youden’s J was adopted as a neutral operating point balancing sensitivity and specificity, given the absence of established clinical cost ratios for false-positive and false-negative decisions in liver graft allocation.

Although Youden’s J provides a balanced operating point under the assumption of symmetric misclassification costs, we acknowledge that clinical decision-making in liver graft allocation is inherently asymmetric, with potentially different consequences for false-positive and false-negative decisions. In the present study, we did not perform a formal sensitivity analysis using alternative thresholds to explicitly model this asymmetry, as there are currently no well-established or consensus-driven cost ratios to parameterize such an approach. This choice was made deliberately to avoid introducing speculative or arbitrarily defined weighting schemes that could bias the interpretation of model performance.

If one error type had to be regarded as relatively more tolerable in this setting, a false negative would be somewhat more acceptable than a false positive. Under the present survival-oriented formulation, a false positive corresponds to proceeding with a donor–recipient match predicted to survive but followed by early posttransplant mortality, which simultaneously exposes a recipient to major surgery and consumes a scarce deceased-donor graft with limited realized benefit. By contrast, a false negative reflects an overly conservative rejection of a potentially viable match; while also undesirable in a system marked by organ scarcity and waiting-list pressure, it does not necessarily imply irreversible harm of the same magnitude, particularly when the model is used only as an auxiliary signal rather than as a stand-alone exclusion rule. For this reason, in the Brazilian context, we consider operating regions with excessive false-positive rates less acceptable than those with a modest degree of overcaution. However, no consensus quantitative cost ratio currently exists.

Future work should explore clinically informed threshold selection strategies, including cost-sensitive analyses or utility-based frameworks, to better align model deployment with real-world decision-making contexts.

To provide a direct comparison between algorithms in the most recent clinical scenario, the predictive performance of each model was evaluated using the last temporal window of the sliding validation framework, in which models were trained on transplant data from 2018 to 2021 and evaluated on the held-out 2022 cohort.

Table 3 summarizes the discrimination and classification performance metrics obtained for each model. The prevalence of recipients surviving beyond 12 months in the test cohort was 79.6%, reflecting the known class imbalance of posttransplant survival outcomes.

**Table 3.** Classification performance of predictive models in the final temporal validation window (train: 2018 to 2021; test: 2022)

Model	ROC-AUC	PR-AUC	AUC CI	Prev	Sens	Spec	Prec	Rec	F1
XGBoost	0.588	0.836	[0.521–0.645]	0.796	0.765	0.415	0.836	0.765	0.799
Random forest	0.595	0.842	[0.527–0.660]	0.796	0.708	0.489	0.844	0.708	0.770
SVM	0.593	0.840	[0.525–0.654]	0.796	0.713	0.457	0.837	0.713	0.770
Logistic regression	0.599	0.842	[0.529–0.663]	0.796	0.656	0.543	0.848	0.656	0.740
HGBM	0.579	0.831	[0.514–0.643]	0.796	0.612	0.553	0.842	0.612	0.709
Current updated MELD (survival)	0.558	0.823	[0.489–0.613]	0.796	0.863	0.255	0.819	0.863	0.840

ROC-AUC, receiver operating characteristic–area under the curve; PR-AUC, precision–recall area under the curve; CI, confidence interval; Prev, prevalence; Sens, sensitivity; Spec, specificity; Prec, precision; Rec, recall; SVM, support vector machine; MELD, Model for End-Stage Liver Disease; HGBM, histogram-based gradient boosting machine

Among all evaluated models, logistic regression achieved the highest ROC-AUC (0.599), followed closely by random forest (0.595) and SVM (0.593). These values indicate a moderate discriminative capacity to distinguish recipients who survive beyond 12 months after transplantation from those who do not. The substantial overlap between confidence intervals suggests that differences in ROC-AUC across models are not statistically significant.

However, because the dataset exhibits a high prevalence of survival, the PR-AUC provides a more informative evaluation of predictive performance. In this metric, logistic regression and random forest again demonstrated the best performance (PR-AUC = 0.842), followed by SVM (0.840), suggesting a limited but consistent ability to identify recipients likely to survive beyond 12 months. Given that survival is the majority class, PR-AUC values are naturally elevated and should be interpreted alongside ROC-AUC to avoid overly optimistic conclusions.

From a clinical perspective, false positives correspond to patients predicted to survive who may experience early mortality, which may lead to overestimation of transplant benefit.

From a threshold-based perspective, XGBoost achieved the highest sensitivity (0.765), indicating the strongest ability to identify recipients who survive beyond 12 months correctly. Higher sensitivity indicates a greater ability to identify surviving patients, although this may come at the cost of reduced specificity and more false-positive predictions. However, this improvement occurred at the expense of specificity (0.415), meaning that the model tends to classify many cases as positive. This behavior may be relevant in clinical contexts where overestimating survival could lead to suboptimal risk stratification.

While XGBoost achieved the highest F1-score (0.799), this metric reflects performance on the majority class (survival) and may mask reduced performance in identifying high-risk patients.

#### Current updated MELD comparison

The performance of the current updated MELD was examined as a clinical reference baseline within the same temporal validation framework used for the machine-learning models. Because MELD was originally formulated to estimate mortality risk rather than calibrated 12-month posttransplant survival probabilities, its values were inverted so that higher transformed values corresponded to higher expected survival, ensuring consistency with the study outcome definition (≥12-month survival). The transformed scores were then min–max normalized to enable rank-preserving comparison and exploratory threshold-based benchmarking alongside the multivariable models. Accordingly, comparisons involving discrimination metrics are more direct than comparisons involving calibration- and decision-analytic metrics, which should be interpreted as approximate because the transformed MELD values do not constitute a formally recalibrated survival model for this cohort. Under this exploratory benchmarking strategy, current updated MELD showed high sensitivity but limited specificity, supporting its role as a clinically familiar reference while also illustrating its limitations as a stand-alone predictor of posttransplant survival in this dataset.

The current updated MELD demonstrated very high sensitivity (0.863) but low specificity (0.255), indicating a tendency to classify most patients as likely to survive. This highlights its utility as a conservative clinical baseline but also its limited discriminative capacity.

Overall, all models demonstrated modest discriminative performance, indicating that predicting posttransplant survival remains a challenging task even with advanced machine-learning approaches.

### Calibration and decision-analytic analysis

Beyond discrimination performance, evaluating the reliability of predicted probabilities is essential in clinical decision-support systems. For this purpose, model calibration was assessed using the Brier score, Brier skill score (BSS), and the Hosmer–Lemeshow (HL) goodness-of-fit test. In addition, decision-analytic performance was assessed using DCA, with the maximum and mean net benefit values.

The Brier score measures the accuracy of probabilistic predictions by calculating the mean squared difference between predicted probabilities and observed outcomes. It is defined as

$$\text{Brier}=\frac{1}{N}\sum_{i=1}^{N}{\left(p_{i}-y_{i}\right)}^{2}$$ (4)

where $p_{i}$ represents the predicted probability for observation i, $y_{i}$ represents the true outcome (0 or 1), and N is the total number of observations. Lower Brier values indicate better probabilistic calibration.

The Brier baseline represents the Brier score obtained from a noninformative model that predicts the same probability for all observations, equal to the prevalence of the positive class in the dataset. It serves as a reference point for evaluating whether a predictive model provides meaningful improvement over a naive strategy.

BSS quantifies the relative improvement of a predictive model compared with the baseline model. It is calculated as

$$\mathrm{BSS}=1-\frac{{\text{Brier}}_{\text{model}}}{{\text{Brier}}_{\text{baseline}}}$$ (5)

A positive BSS indicates that the model performs better than the baseline predictor, while negative values suggest worse performance.

Calibration was further assessed using the HL goodness-of-fit test, which evaluates whether predicted probabilities are consistent with observed outcome frequencies across risk strata. Observations are typically grouped into deciles of predicted risk, and the HL statistic is computed as

$$\chi_{\mathrm{HL}}^{2}=\sum_{g=1}^{G}\frac{{\left(O_{g}-E_{g}\right)}^{2}}{E_{g}\left(1-E_{g}/n_{g}\right)}$$ (6)

where $O_{g}$ represents the observed number of events in group g, $E_{g}$ represents the expected number of events, and $n_{g}$ is the number of observations in the group.

DCA quantifies the clinical value of a predictive model by calculating the net benefit across a range of threshold probabilities. Net benefit incorporates the trade-off between correctly identified positive outcomes (true positives) and incorrectly accepted cases (false positives), weighted by the clinical consequences of each decision. The net benefit is defined as

$$\mathrm{NB}=\frac{\mathrm{TP}}{N}-\frac{\mathrm{FP}}{N}\left(\frac{p_{t}}{1-p_{t}}\right)$$ (7)

where TP and FP represent the number of true positives and false positives, respectively; N is the total number of observations; and $p_{t}$ is the threshold probability.

This formulation adjusts the penalty for false positives based on the clinical risk tolerance implied by the decision threshold. Higher threshold probabilities indicate a more conservative decision policy, where transplants are accepted only when predicted survival probabilities are sufficiently high.

The DCA framework compares the net benefit of predictive models with 2 baseline clinical strategies. The “treat all” strategy assumes that every donor–recipient pair is accepted for transplantation regardless of predicted survival probability, while the “treat none” strategy assumes that no transplants are performed. A predictive model demonstrates clinical utility when its decision curve lies above both baseline strategies within a clinically relevant range of threshold probabilities.

The corresponding HL *P* value tests the null hypothesis that predicted and observed event rates are statistically consistent. A large *P* value (typically P>0.05) indicates good calibration, while small values suggest significant discrepancies between predicted probabilities and observed outcomes.

Table 4 summarizes these results for the final temporal validation window.

**Table 4.** Calibration and decision-analytic metrics for predictive models in the final temporal validation window (train: 2018 to 2021; test: 2022)

Model	Brier	Brier baseline	Brier skill	HL $\chi^{2}$	HL *P* value	Best net benefit	Mean net benefit
XGBoost	0.160	0.163	0.015	13.386	0.099	0.659	0.361
Random forest	0.159	0.163	0.021	12.116	0.146	0.659	0.366
SVM	0.159	0.163	0.021	3.853	0.870	0.659	0.365
Logistic regression	0.159	0.163	0.025	6.896	0.548	0.659	0.367
HGBM	0.159	0.163	0.023	13.532	0.095	0.659	0.365
Current updated MELD (survival)	0.204	0.163	−0.255	93.547	0.000	0.642	0.242

HL, Hosmer–Lemeshow; SVM, support vector machine; MELD, Model for End-Stage Liver Disease; HGBM, histogram-based gradient boosting machine

The logistic regression model achieved the lowest Brier score (0.159) together with the highest BSS (0.025), indicating the most accurate probabilistic predictions relative to the baseline prevalence model. The histogram-based gradient boosting machine (HGBM) showed similar calibration performance (Brier = 0.159; Brier skill = 0.023), followed closely by SVM and random forest (Brier skill = 0.021).

Calibration quality was further evaluated using the HL test. Logistic regression (HL *P* = 0.548), SVM (HL *P* = 0.870), random forest (HL *P* = 0.146), XGBoost (HL *P* = 0.099), and HGBM (HL *P* = 0.095) showed no evidence of lack of fit, although the limitations of the HL test in large samples should be considered.

DCA indicated a positive net benefit across the evaluated threshold range for all models. However, given the modest discrimination and the exploratory nature of the MELD benchmarking strategy, these findings should be interpreted as comparative evidence of model behavior rather than as direct estimates of clinical effectiveness.

Across models, the differences in net benefit were modest. Although the multivariable models showed more favorable results than the transformed MELD baseline in this cohort, the magnitude of these differences should be interpreted cautiously and should not be taken as sufficient evidence for operational deployment.

Overall, these results indicate that in structured clinical datasets, well-calibrated statistical models can perform comparably to more complex machine-learning approaches while offering advantages in interpretability and calibration stability.

### Minority-class (mortality) performance analysis

Although the primary objective of this study is to predict posttransplant survival, from a clinical perspective, it is equally important to assess the models’ ability to identify high-risk recipients who may not survive within the first 12 months. While the primary analysis focused on predicting survival, this complementary evaluation assesses the models’ ability to identify mortality, a clinically critical minority outcome.

To address this, additional performance metrics were computed, considering mortality (class 0) as the positive class. Table 5 reports the corresponding results for the final temporal validation window.

**Table 5.** Minority-class (mortality) performance in the final temporal validation window (train: 2018 to 2021; test: 2022). Mortality (death) is treated as the positive class.

Model	PR-AUC (death)	Precision	Recall	F1
Logistic regression	0.320	0.288	0.543	0.376
Random forest	0.305	0.301	0.489	0.372
SVM	0.304	0.291	0.457	0.355
HGBM	0.295	0.268	0.553	0.361
XGBoost	0.290	0.312	0.415	0.356
Current updated MELD (death)	0.184	0.000	0.000	0.000

PR-AUC, precision–recall area under the curve; SVM, support vector machine; MELD, Model for End-Stage Liver Disease; HGBM, histogram-based gradient boosting machine

Overall, the results indicate that predictive performance for the minority class is substantially lower than that for the survival class. In the final temporal validation window, PR-AUC values ranged from 0.184 to 0.320, reflecting the difficulty of identifying mortality events in an imbalanced setting.

In this final-window comparison, logistic regression achieved the highest PR-AUC (0.320), whereas HGBM achieved the highest recall (0.553). Across all machine-learning models, precision remained relatively low, highlighting the difficulty of identifying mortality cases without introducing a substantial number of false positives.

This behavior reflects both the intrinsic class imbalance and the difficulty of learning consistent patterns associated with adverse outcomes. The observed results suggest that mortality prediction remains highly sensitive to threshold selection and model specification.

From a clinical perspective, because death is treated as the positive class in this subsection, the interpretation of false positives and false negatives is reversed relative to the survival-oriented formulation used in the “Model performance in the final temporal window (2018 to 2021 → 2022)” section. False negatives—patients predicted to survive who die within 12 months—represent the most critical error, as they correspond to potentially avoidable adverse outcomes. Conversely, false positives may lead to unnecessary rejection of viable transplant opportunities. This trade-off is evident across temporal windows, reinforcing the need for careful threshold selection. The confusion matrix results (Table A7) illustrate this trade-off, with some windows showing a large number of false negatives and others exhibiting excessive false positives.

These findings highlight an important trade-off: while the models demonstrate consistent ability to identify patients likely to survive, their capacity to detect high-risk cases remains limited. This performance asymmetry is expected in imbalanced clinical datasets and reinforces the need for cautious interpretation when applying such models in decision-support settings.

This behavior is consistent with the moderate discrimination performance observed across models, further emphasizing the complexity of predicting posttransplant mortality, and is consistent with the DCA results, which capture the clinical consequences of this trade-off across different decision thresholds.

In Table 5, logistic regression achieved the highest PR-AUC (0.320), indicating the highest (although still limited) discrimination for identifying high-risk recipients among the evaluated models. In contrast, HGBM obtained the highest recall (0.553), followed by logistic regression (0.543) and random forest (0.489), demonstrating moderate sensitivity to mortality events, albeit at the expense of low precision.

XGBoost showed the highest precision among the evaluated models (0.312), reflecting a more conservative profile with fewer positive mortality predictions. Overall, however, minority-class performance remained limited across all models, with low precision and PR-AUC values at or below 0.320, indicating the difficulty of capturing mortality events consistently. Across all machine-learning models, precision remained relatively low (approximately 0.26 to 0.30), highlighting the inherent difficulty of accurately identifying mortality cases without introducing a substantial number of false positives.

The current version of the MELD mortality baseline failed to identify any mortality cases in this evaluation setting (recall = 0.000), resulting in a precision and F1-score of 0.000. This behavior reflects its limitation as a stand-alone predictor when evaluated under a mortality-focused framework.

Overall, predictive performance for the minority class (mortality) was substantially lower and more variable than that for survival.

### Global model simulation and decision-analytic metrics

To provide an additional retrospective decision-analytic perspective, we conducted a full-cohort counterfactual simulation using all transplants performed between 2007 and 2022 and the models trained on the most recent temporal window. This analysis does not represent prospective deployment, observed clinical decision-making, or measured intervention effects. Instead, it provides an exploratory estimate of how different predictive models would compare if used as auxiliary decision-support signals under the assumptions of the present retrospective framework.

The evaluation of this scenario was conducted using probabilistic calibration metrics and decision-analytic analysis through DCA.

**Fig. 3.**
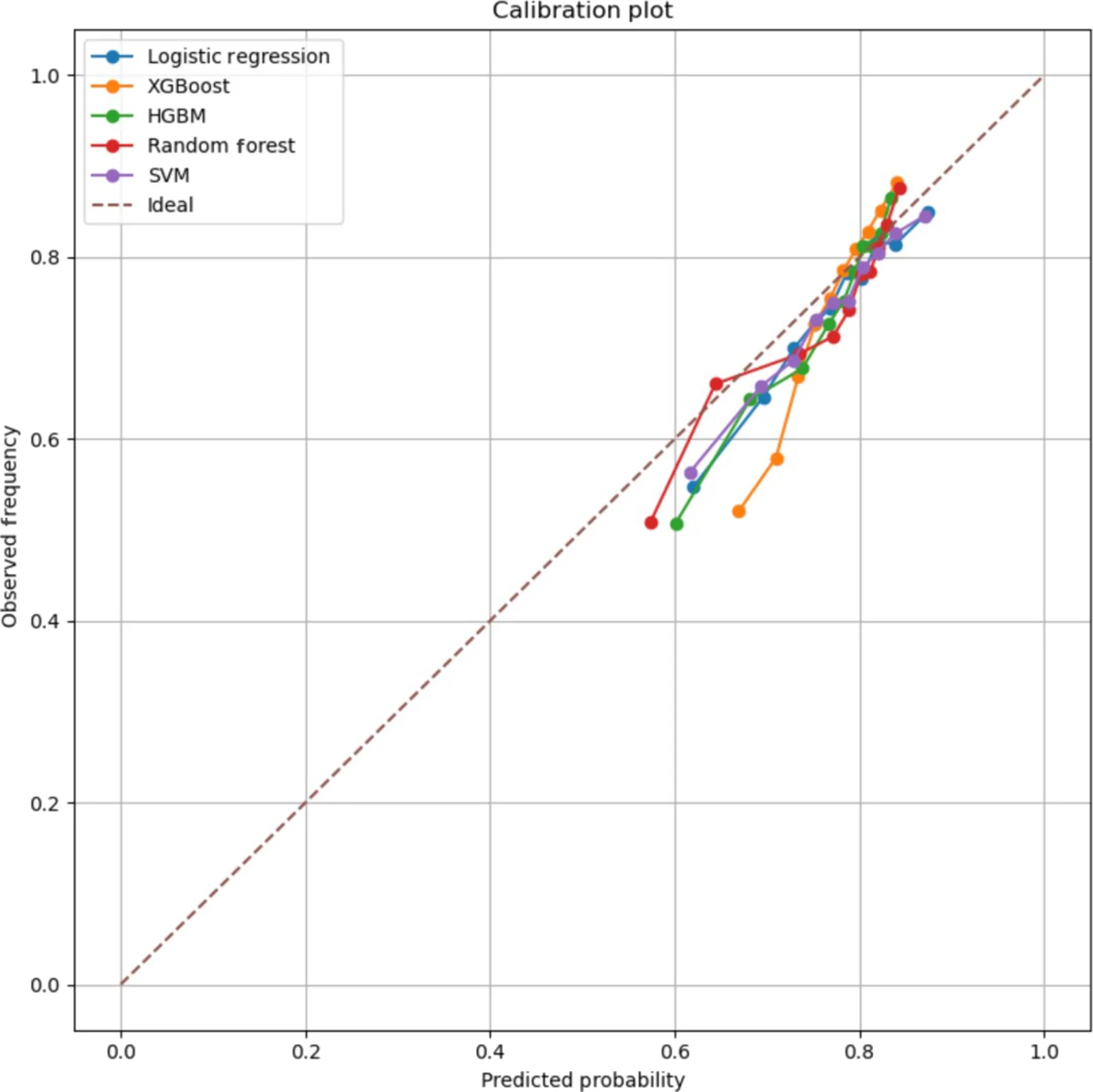
Platt calibration plot 2007 to 2022.

The calibration plot shown in Fig. 3 illustrates the agreement between predicted probabilities and observed outcome frequencies for all evaluated models. Minor deviations from the ideal calibration line are observed, particularly in the intermediate-probability range (0.6 to 0.7). Performance appeared more stable at higher predicted probabilities (>0.75), which are more relevant for clinical decision-making.

**Fig. 4.**
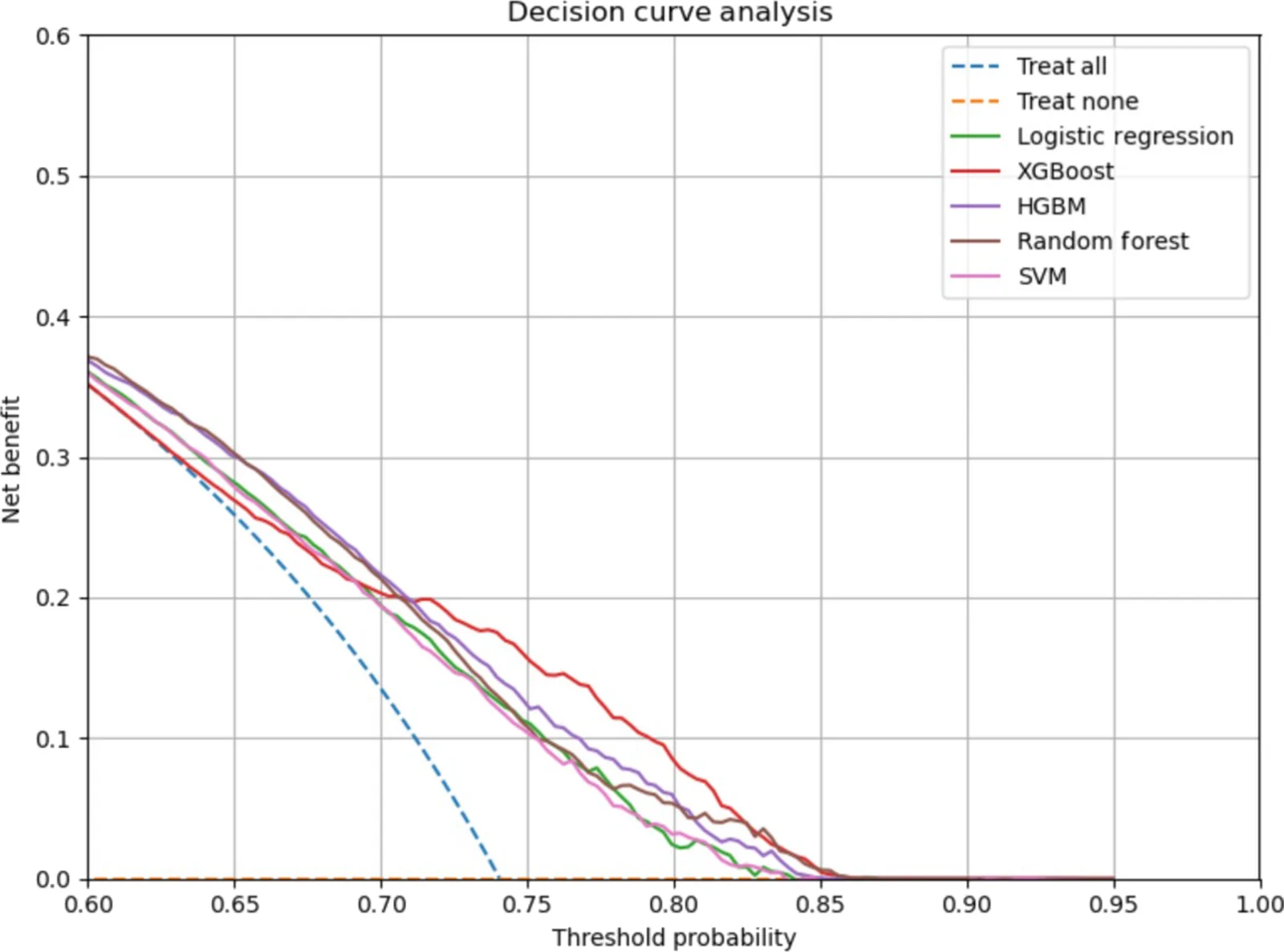
Decision curve analysis (2007 to 2022).

To evaluate comparative decision-analytic behavior across models, DCA was performed, as illustrated in Fig. 4. Unlike traditional discrimination metrics such as ROC-AUC or PR-AUC, which measure a model’s ability to separate classes, DCA evaluates the expected decision consequences of using a model across threshold probabilities.

Figure 4 presents the decision curves for all evaluated models. The vertical axis represents the net benefit obtained by applying each model, while the horizontal axis represents the threshold probability used for decision-making. Models that achieve higher net benefit values across the relevant threshold range indicate more favorable decision-analytic performance within the explored retrospective framework, rather than directly observed improvements in real allocation outcomes.

In this study, the threshold range of 0.60 to 0.85 was used as an exploratory range to represent plausible posttransplant risk tolerances in a high-stakes allocation context. This range was not derived from a formally elicited clinical utility model and should therefore be interpreted as a comparative analytical setting rather than as a definitive operational decision rule. Within this exploratory range, all evaluated models showed a positive net benefit relative to default strategies. From a decision-analytic perspective, the observed net benefit differences suggest a modest potential gain under the evaluated threshold range. However, these estimates are based on a retrospective counterfactual framework and should not be interpreted as direct clinical gains observed in prospective deployment.

Table 6 shows that, overall, HGBM achieved the highest net benefit at a reference threshold of 0.70 (NB_0.70_ = 0.216), whereas XGBoost showed the highest average net benefit across the broader clinically relevant range (NB_0.60–0.85_ = 0.180). These differences should be interpreted as indicating modest decision-analytic variation across models within the retrospective framework adopted here, rather than as directly observed clinical gains under prospective use.

**Table 6.** Decision-analytic and calibration performance in the full-cohort simulation (2007 to 2022)

Model	Brier	NB_0.60–0.85_	NB_0.70_
HGBM	0.183	0.178	0.216
Random forest	0.183	0.175	0.214
XGBoost	0.184	0.180	0.203
Logistic regression	0.186	0.160	0.195
SVM	0.186	0.158	0.194

NB_0.60–0.85_, mean net benefit in the clinically relevant threshold range (0.60 to 0.85); NB_0.70_: net benefit at threshold probability 0.70; SVM, support vector machine; HGBM, histogram-based gradient boosting machine

Random forest demonstrated comparable performance across both metrics, whereas logistic regression and SVM showed slightly lower yet still consistent net benefit values. Despite these differences, the magnitude of variation across models was small, indicating broadly similar decision-analytic performance among the evaluated approaches.

These findings are consistent with the modest discrimination and calibration performance observed in the temporal validation analysis, reinforcing the complexity of predicting posttransplant survival.

### Most important features analysis

To better understand how the models estimate posttransplant survival, we analyzed the most influential predictors identified by each algorithm and examined their consistency, clinical interpretability, and potential redundancy.

Table 7 summarizes the top 10 predictors identified by each model using model-specific feature-importance measures. For tree-based models, feature importance was estimated using Shapley additive explanations (SHAP)-based values, whereas standardized coefficients were used for logistic regression. Across all algorithms, current updated MELD consistently emerged as the dominant predictor. This finding reinforces the central role of liver disease severity in determining posttransplant outcomes and aligns with the established clinical use of MELD in organ allocation.

**Table 7.** Top 10 features by model-specific importance (SHAP gain for XGBoost/gradient boosting; standardized coefficients for logistic regression)

Model	Important variables
Logistic regression	Current updated MELD, recipient height, recipient age at transplant, diagnosis, anti-HBc (record), donor pO_2_, recipient anti-HBs, recipient cold ischemia time, organ retrieval time, total ischemia time
XGBoost	Current updated MELD, hematocrit (HCT), total ischemia time, donor AST (aspartate aminotransferase), donor GGT (gamma-glutamyl transferase), recipient weight, leukocyte count, diagnosis, donor blood glucose, donor creatinine
SVM	Current updated MELD, diagnosis, recipient height, donor pO_2_, recipient age at transplant, anti-HBc (record), recipient cold ischemia time, organ retrieval time, total ischemia time, white blood cell count
Random forest	Current updated MELD, recipient height, diagnosis, total ischemia time, donor CPK (creatine phosphokinase), organ retrieval time, white blood cell count (leukocytes), recipient age at transplant, donor pCO_2_, donor pO_2_
HGBM	Current updated MELD, total ischemia time, recipient height, recipient race, anti-HBc (record), recipient weight, donor creatinine, recipient age at transplant, donor sodium, urea

SHAP, Shapley additive explanations; MELD, Model for End-Stage Liver Disease; SVM, support vector machine; HGBM, histogram-based gradient boosting machine; anti-HBc, antibody to hepatitis B core antigen; anti-HBs, antibody to hepatitis B surface antigen

Beyond MELD-related predictors, several recipient, donor, and procedural variables were consistently identified across models. Recipient age at transplant showed a stable negative association with survival, which is clinically coherent and supported by prior evidence indicating increased posttransplant risk among older patients. Similarly, transplant-related factors such as total ischemia time, recipient cold ischemia time, and procurement time were negatively associated with survival, reflecting the well-established impact of ischemic injury on graft function.

Donor-related physiological variables, including donor pO_2_, were also identified as relevant predictors. This may reflect aspects of donor stability and organ viability; however, the magnitude of these effects was modest, and their clinical interpretation remains indirect.

Some variables identified by the models require more cautious interpretation. For example, recipient height appeared as a positive predictor of survival. While this may be partially explained by donor–recipient size matching, it may also reflect underlying demographic or physiological factors not explicitly modeled. Similarly, leukocyte count and anti-HBc (antibody to hepatitis B core antigen) status were associated with improved predicted survival. Still, these relationships are not straightforward from a clinical perspective and may reflect residual confounding, selection effects, or proxy relationships.

To assess the robustness of feature importance across algorithms, a stability analysis was performed (Table 8). MELD-related variables and diagnosis were consistently identified across all models, while recipient age, recipient height, anti-HBc status, and donor pO_2_ also showed high recurrence. This consistency across models suggests that these variables represent robust predictive signals rather than model-specific artifacts.

**Table 8.** Feature stability across models. Variables are ranked in descending order according to the number of models in which they appear among the top predictors.

Feature	Models identifying feature
Current updated MELD	5
Total ischemia time	5
Recipient height	4
Diagnosis	4
Recipient age at transplant	4
White blood cell count (leukocytes)	3
Organ retrieval time	3
Donor pO_2_	3
Anti-HBc (record)	3
Recipient weight	2
Donor creatinine	2
Recipient cold ischemia time	2
Recipient anti-HBs	1
Hematocrit (HCT)	1
Donor AST (aspartate aminotransferase)	1
Donor GGT (gamma-glutamyl transferase)	1
Donor blood glucose	1
Donor CPK (creatine phosphokinase)	1
Donor pCO_2_	1
Recipient race	1
Donor sodium	1
Urea	1

MELD, Model for End-Stage Liver Disease; anti-HBc, antibody to hepatitis B core antigen; anti-HBs, antibody to hepatitis B surface antigen

For interpretability, logistic regression coefficients were analyzed to assess the direction of association with survival probability (Table 9). Higher current updated MELD, older recipient age, diagnosis category, donor pO_2_, and ischemia-related variables (cold, procurement, and total ischemia time) were associated with reduced survival. In contrast, recipient height, anti-HBc status, and anti-HBs (antibody to hepatitis B surface antigen) were associated with increased survival. These relationships should be interpreted as associative rather than causal, particularly when a residual correlation exists among clinically related predictors.

**Table 9.** Top 10 most influential features in the logistic regression model (final temporal validation window). The sign of the coefficient indicates the direction of the effect on the predicted survival probability.

Feature	Coefficient	Direction
Current updated MELD	−0.3186	Decreases survival
Recipient height	0.2017	Increases survival
Recipient age at transplant	−0.1948	Decreases survival
Diagnosis	−0.1922	Decreases survival
Anti-HBc (record)	0.1687	Increases survival
Donor pO_2_	−0.1409	Decreases survival
Recipient anti-HBs	0.1250	Increases survival
Recipient cold ischemia time	−0.1185	Decreases survival
Procurement time	−0.1154	Decreases survival
Total ischemia time	−0.1154	Decreases survival

MELD, Model for End-Stage Liver Disease; anti-HBc, antibody to hepatitis B core antigen; anti-HBs, antibody to hepatitis B surface antigen

It is important to note that feature importance derived from SHAP values (for tree-based models) and standardized coefficients (for logistic regression) are not directly comparable, as they capture different aspects of model behavior.

Importantly, feature importance does not imply causality. The observed associations may reflect indirect effects, proxy relationships, or dataset-specific patterns rather than direct physiological mechanisms. Therefore, these findings should be interpreted as hypothesis generating rather than causal evidence.

Overall, this analysis indicates that the models rely primarily on clinically recognizable predictors—particularly those related to liver disease severity and transplant logistics—while also identifying additional variables that may contribute to risk stratification. However, careful interpretation is required, especially in the presence of correlated features, indirect associations, and complex clinical interactions.

Taken together, these findings suggest that model predictions are driven primarily by recognizable severity and logistics variables. In contrast, the contributions of several secondary predictors are indirect and should be interpreted as hypothesis generating.

#### SHAP dependence analysis and clinical interpretation

To further investigate the relationship between selected clinically relevant predictors and posttransplant survival, Appendix E presents SHAP dependence plots derived from the Logistic Regression model.

Logistic regression was selected for interpretability analysis because of its competitive performance and relatively transparent structure compared with other models.

These dependence plots illustrate the marginal effect of each selected variable on the predicted survival probability within this specific modeling framework while also providing insight into the direction and magnitude of the effect, as well as potential nonlinearities and interaction patterns with baseline disease severity (current updated MELD).

The relationships observed in several variables were predominantly monotonic and approximately linear, suggesting that model predictions are largely driven by additive effects rather than complex nonlinear interactions.

The SHAP dependence analysis for the current updated MELD shows a strong monotonic association with predicted posttransplant survival, with higher values associated with lower predicted survival probability. In this model, the decline becomes more pronounced in the upper range of the standardized MELD values. This pattern is broadly consistent with the established clinical meaning of MELD as a marker of disease severity. However, the dependence plot should be interpreted as a model-based descriptive pattern rather than as evidence of a biologically validated threshold.

The SHAP dependence plot for diagnosis suggests heterogeneous associations across diagnostic categories. Because diagnoses were encoded categorically and projected into a model-specific numerical representation, these patterns should be interpreted with caution. They should not be read as evidence of an ordinal biological gradient. At most, they indicate that the underlying indication for transplantation contributes to the model’s risk estimates in a nonuniform manner.

The SHAP dependence analysis for donor ABO shows relatively small variations in SHAP values, indicating a limited direct contribution to predicted survival within this model. Because ABO compatibility is already a prerequisite for allocation, these differences are better interpreted as residual model-level associations than as evidence of a prognostic threshold or an independent biological effect. In this sense, donor ABO should be viewed primarily as a feasibility constraint in allocation logistics rather than as a major driver of posttransplant prognosis in the present modeling framework.

The SHAP dependence plot for prioritization shows a negative association with predicted survival. This pattern is consistent with the observation that higher-priority patients generally present with greater clinical severity, including conditions not fully captured by MELD alone. However, because prioritization is a policy and clinically mediated variable and because ordinal encoding may impose monotonic structure, this pattern should be interpreted cautiously as a descriptive model-level association rather than as evidence of a validated prognostic threshold.

Recipient height emerged as a recurrent predictor across models, but its interpretation remains indirect. This variable may partially reflect donor–recipient size compatibility or other unmeasured clinical and demographic correlates. Accordingly, its contribution should be interpreted as associative and hypothesis generating rather than as a direct physiological determinant of posttransplant survival.

Taken together, these results indicate that the models rely primarily on clinically recognizable severity and logistics variables while also highlighting additional predictors whose interpretation remains indirect and should be treated as hypothesis generating.

The near-linear patterns observed in variables such as diagnosis and prioritization may reflect the ordinal encoding of categorical variables, which can impose artificial monotonic relationships in SHAP dependence plots.

## Conclusion

Since the pioneering liver transplant performed by surgeon Thomas Starzl in 1963, this procedure has saved many lives worldwide. Over the decades, advances in surgical techniques and immunosuppressive therapies have led to substantial improvements in patient outcomes, with approximately 85% of recipients surviving beyond the first year. Nevertheless, the persistent shortage of donor organs, combined with the inherent subjectivity of graft acceptance decisions and the expansion of donor criteria, continues to pose a major challenge in modern transplantation systems.

In this context, the study suggests that machine-learning models trained on routinely available donor–recipient variables can provide complementary decision-support information for predicting 12-month posttransplant survival. Using a large real-world transplant registry from the state of São Paulo and a temporally structured validation framework, multiple algorithms were evaluated under conditions that approximate prospective clinical deployment.

Although discrimination performance remained modest, the evaluated models showed comparatively more stable probabilistic calibration and positive net benefit within the explored decision-threshold range. In the present retrospective framework, they also generally exceeded the transformed MELD baseline in decision-analytic comparisons, suggesting that donor–recipient interaction variables may provide additional prognostic information beyond traditional severity scores.

These results reinforce that in this setting, model assessment should not rely exclusively on discrimination metrics. Calibration- and decision-analytic measures provided additional information about model behavior, although the overall predictive performance remained modest and the mortality-focused results were unstable.

Among the evaluated approaches, logistic regression showed the most consistent balance across discrimination, calibration, and net benefit, but differences across models were small. Accordingly, the present findings should be interpreted as evidence that routinely collected donor–recipient variables contain a limited but nonnegligible prognostic signal, rather than as evidence that any single model is ready for stand-alone clinical deployment.

From a translational perspective, the study supports further investigation of calibrated machine-learning models as complementary decision-support tools. However, external validation, explicit threshold-sensitive evaluation, and prospective clinical assessment remain necessary before any operational use in allocation practice can be justified.

## Limitations and Future Directions

### Limitations

First, the models were developed using retrospective registry data from a single geographic region (the state of São Paulo, Brazil). Although the dataset represents one of the largest liver transplant registries in Latin America, variations in donor characteristics, healthcare infrastructure, and clinical practices may limit the generalizability of the models to other transplant systems. Nevertheless, São Paulo has a highly heterogeneous population shaped by intense internal migration and long-standing international immigration, resulting in considerable ethnic and sociodemographic diversity within the cohort. This feature may partially mitigate concerns regarding representativeness, but it does not remove the need for external validation. Although temporal validation was performed to mitigate potential temporal drift, external validation using datasets from other geographic regions will still be necessary to confirm the generalizability of the proposed models.

Second, another important limitation is that clinical teams historically determined transplant acceptance decisions in the registry. Consequently, the dataset may implicitly reflect clinical selection biases regarding which donor–recipient combinations were ultimately performed. These historical decision patterns may influence the statistical relationships learned by the predictive models. Although the predictive models showed positive net benefit within the explored threshold range and comparatively stable probabilistic calibration, the overall discrimination performance remained moderate. This reflects the inherent complexity of predicting posttransplant survival using registry variables alone, where many relevant biological and perioperative factors may not be fully captured in structured datasets.

Third, the available dataset did not include several potentially relevant clinical variables, such as detailed graft histology, intraoperative surgical variables, or center-specific decision policies. The absence of these factors may limit the maximum achievable predictive performance.

Fourth, although temporal validation was performed to simulate real-world deployment, the models were not prospectively evaluated in a clinical environment. Consequently, the estimated clinical impact derived from DCA and simulation experiments should be interpreted as a theoretical approximation rather than a confirmed clinical benefit.

Fifth, performance in the minority class (mortality) was substantially lower and unstable across temporal windows, reflecting the intrinsic difficulty of predicting adverse outcomes in imbalanced datasets.

Sixth, although redundant MELD-derived variables were excluded from the final models to reduce redundancy, residual correlation among clinically related severity and transplant logistics variables may still influence coefficient stability and limit the interpretation of individual predictors. Therefore, feature importance should be interpreted as associative rather than causal.

Finally, the models were evaluated using routinely available variables at the time of transplant decision, which may not capture all relevant clinical nuances considered by transplant teams.

An additional limitation is that the MELD comparison required score inversion and normalization to align the original mortality-oriented scale with the survival-oriented study endpoint. While this enabled exploratory benchmarking, it does not constitute a formal recalibration of MELD for 12-month posttransplant survival. It therefore limits the strength of calibration and decision-analytic comparisons involving this baseline.

Moreover, although complete-case analysis was adopted to avoid imputation-related assumptions in key prognostic variables, this choice may have introduced selection effects if excluded cases differed systematically from included cases. Therefore, the resulting cohort should be interpreted as an analytically filtered subset of the registry rather than as a fully unbiased representation of all eligible transplants.

These limitations highlight the importance of cautious interpretation and emphasize the need for prospective clinical validation before the models can be considered for routine clinical use.

### Future directions

One important avenue involves external validation using transplant registries from other geographic regions or international databases such as the United Network for Organ Sharing or Eurotransplant. Such analyses would help determine the robustness and generalizability of the predictive models across different healthcare systems.

Another promising direction involves integrating additional clinical information sources, including imaging data, histopathological findings, and intraoperative variables. Multimodal models combining structured clinical data with imaging or genomic information may improve predictive performance and capture more complex patterns associated with graft survival. In this context, future progress may also benefit from more integrated computational frameworks that combine heterogeneous data sources and model multiple process components in a coordinated manner, an approach emphasized in recent *Computational and Structural Biotechnology Journal* work on multistage and multicomponent computational modeling in regenerative medicine and biofabrication [22].

Future work should also explore advanced explainability techniques within the field of explainable AI. Improving the interpretability of predictive models is essential for increasing clinician trust and facilitating the integration of AI-assisted decision-support tools into transplant practice.

Future research should focus on improving the prediction of adverse outcomes, particularly mortality, by developing models optimized for the minority class or by using alternative formulations such as survival analysis or time-to-event modeling.

Finally, prospective clinical studies will be necessary to evaluate how AI-assisted predictions influence real-world clinical decisions. Controlled trials or observational deployment studies could measure whether the use of calibrated predictive models improves graft utilization, reduces organ discard, or enhances long-term patient outcomes.

Continued collaboration between clinicians, transplant surgeons, and data scientists will be essential to ensure that AI technologies are implemented safely, ethically, and effectively within transplant medicine.

Future research may also explore optimization frameworks that combine survival prediction models with donor–recipient matching algorithms. Such approaches could support allocation strategies that simultaneously consider predicted survival probability and organ utilization efficiency, potentially improving decision-making in transplant allocation systems.

## Figures and Tables

**Fig. F1.**
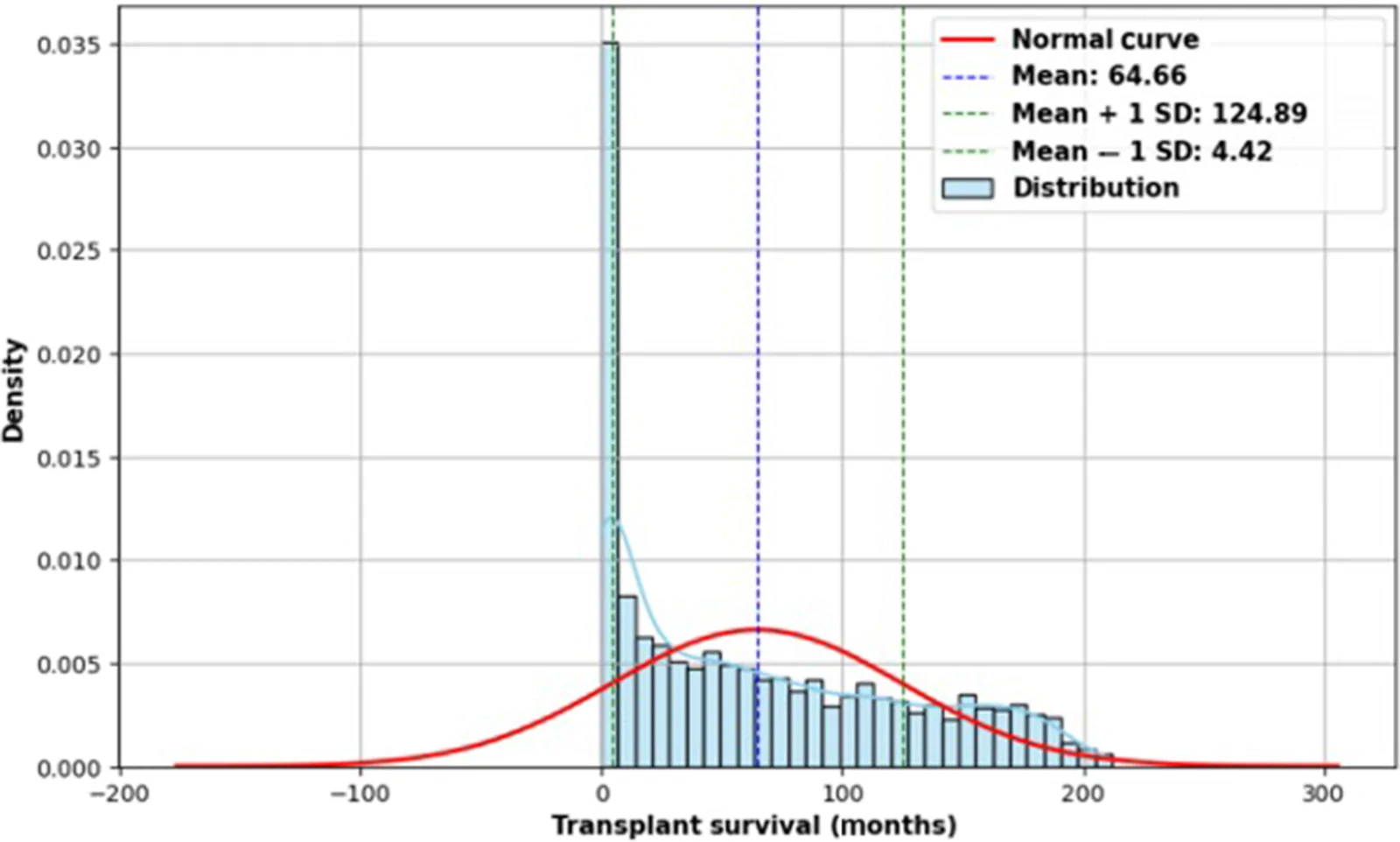
Distribution of posttransplant survival (months) in the São Paulo cohort. Mean = 64.66 months; SD = 60.24 months; 26.71% mortality by 12 months.

## Data Availability

The data supporting the findings of this study are available through a controlled-access Zenodo archive at https://zenodo.org/records/18189988 [23]. Access can be requested through the Zenodo record. The dataset is fully anonymized and was shared in compliance with applicable ethical and data protection requirements. The code used in this study for data preprocessing, model implementation, training, and evaluation is available through a controlled-access Zenodo archive at https://zenodo.org/records/18213728 [20]. Access can be requested through the Zenodo record. The archive contains the scripts required to reproduce the analyses reported in this article.

## Appendix A. Model Metrics Across Temporal Validation

**Table A1.** Logistic regression results across sliding temporal validation windows

Window	Train	Test	roc_auc	auc_ci	pr_auc	Brier	brier_baseline	brier_skill	Prevalence	thr_youden	nb_max	nb_mean	Sens	Spec	Prec	Recall	F1	TN	FP	FN	TP
1	2007–2010	2011	0.708	[0.655–0.756]	0.837	0.189	0.206	0.082	0.710	0.746	0.517	0.260	0.566	0.791	0.869	0.566	0.686	110	29	148	193
2	2008–2011	2012	0.679	[0.626–0.728]	0.860	0.175	0.189	0.075	0.746	0.795	0.582	0.311	0.382	0.886	0.908	0.382	0.538	93	12	191	118
3	2009–2012	2013	0.616	[0.558–0.675]	0.825	0.180	0.184	0.022	0.757	0.708	0.592	0.291	0.591	0.645	0.838	0.591	0.693	71	39	140	202
4	2010–2013	2014	0.611	[0.556–0.671]	0.768	0.199	0.205	0.029	0.712	0.724	0.521	0.217	0.567	0.659	0.805	0.567	0.665	83	43	135	177
5	2011–2014	2015	0.618	[0.557–0.676]	0.794	0.188	0.195	0.034	0.734	0.747	0.557	0.258	0.521	0.691	0.823	0.521	0.638	85	38	163	177
6	2012–2015	2016	0.603	[0.529–0.672]	0.811	0.178	0.183	0.023	0.760	0.703	0.597	0.295	0.713	0.500	0.819	0.713	0.762	49	49	89	221
7	2013–2016	2017	0.588	[0.525–0.641]	0.796	0.183	0.188	0.025	0.749	0.727	0.582	0.277	0.690	0.445	0.788	0.690	0.736	53	66	110	245
8	2014–2017	2018	0.616	[0.558–0.680]	0.842	0.170	0.174	0.025	0.775	0.745	0.626	0.326	0.559	0.637	0.842	0.559	0.672	72	41	172	218
9	2015–2018	2019	0.661	[0.610–0.713]	0.839	0.173	0.183	0.050	0.760	0.779	0.599	0.315	0.519	0.773	0.878	0.519	0.652	92	27	181	195
10	2016–2019	2020	0.627	[0.564–0.681]	0.843	0.169	0.177	0.044	0.771	0.801	0.618	0.331	0.442	0.775	0.869	0.442	0.586	100	29	242	192
11	2017–2020	2021	0.597	[0.534–0.662]	0.838	0.165	0.167	0.014	0.788	0.771	0.647	0.347	0.557	0.639	0.852	0.557	0.673	62	35	160	201
12	2018–2021	2022	0.599	[0.529–0.663]	0.842	0.158	0.163	0.025	0.796	0.756	0.659	0.367	0.656	0.543	0.848	0.656	0.740	51	43	126	240

Sens, sensitivity; Spec, specificity; Prec, precision; TN, true negative; FP, false positive; FN, false negative; TP, true positive

**Table A2.** XGBoost results across sliding temporal validation windows

Window	Train	Test	roc_auc	auc_ci	pr_auc	Brier	brier_baseline	brier_skill	Prevalence	thr_youden	nb_max	nb_mean	Sens	Spec	Prec	Recall	F1	TN	FP	FN	TP
1	2007–2010	2011	0.632	[0.577–0.687]	0.788	0.198	0.206	0.035	0.710	0.726	0.517	0.224	0.595	0.619	0.793	0.595	0.680	86	53	138	203
2	2008–2011	2012	0.648	[0.589–0.707]	0.838	0.180	0.189	0.051	0.746	0.711	0.577	0.292	0.709	0.552	0.823	0.709	0.762	58	47	90	219
3	2009–2012	2013	0.616	[0.554–0.677]	0.834	0.180	0.184	0.022	0.757	0.758	0.594	0.292	0.398	0.791	0.855	0.398	0.543	87	23	206	136
4	2010–2013	2014	0.579	[0.520–0.638]	0.779	0.203	0.205	0.010	0.712	0.773	0.521	0.210	0.378	0.762	0.797	0.378	0.513	96	30	194	118
5	2011–2014	2015	0.637	[0.584–0.693]	0.810	0.186	0.195	0.047	0.734	0.692	0.557	0.268	0.756	0.463	0.796	0.756	0.775	57	66	83	257
6	2012–2015	2016	0.608	[0.545–0.678]	0.813	0.177	0.183	0.029	0.760	0.643	0.600	0.299	0.897	0.316	0.806	0.897	0.849	31	67	32	278
7	2013–2016	2017	0.589	[0.532–0.644]	0.801	0.184	0.188	0.020	0.749	0.691	0.582	0.275	0.848	0.328	0.790	0.848	0.818	39	80	54	301
8	2014–2017	2018	0.551	[0.491–0.609]	0.812	0.176	0.174	−0.009	0.775	0.783	0.626	0.305	0.264	0.858	0.866	0.264	0.405	97	16	287	103
9	2015–2018	2019	0.664	[0.606–0.722]	0.839	0.174	0.183	0.046	0.760	0.748	0.599	0.312	0.652	0.622	0.845	0.652	0.736	74	45	131	245
10	2016–2019	2020	0.605	[0.550–0.661]	0.830	0.172	0.177	0.027	0.771	0.793	0.618	0.320	0.495	0.659	0.830	0.495	0.620	85	44	219	215
11	2017–2020	2021	0.544	[0.478–0.610]	0.809	0.166	0.167	0.003	0.788	0.778	0.647	0.337	0.579	0.526	0.820	0.579	0.679	51	46	152	209
12	2018–2021	2022	0.588	[0.521–0.645]	0.836	0.160	0.163	0.015	0.796	0.744	0.659	0.361	0.765	0.415	0.836	0.765	0.799	39	55	86	280

Sens, sensitivity; Spec, specificity; Prec, precision; TN, true negative; FP, false positive; FN, false negative; TP, true positive

**Table A3.** Random forest results across sliding temporal validation windows

Window	Train	Test	roc_auc	auc_ci	pr_auc	Brier	brier_baseline	brier_skill	Prevalence	thr_youden	nb_max	nb_mean	Sens	Spec	Prec	Recall	F1	TN	FP	FN	TP
1	2007–2010	2011	0.695	[0.645–0.744]	0.841	0.191	0.206	0.071	0.710	0.718	0.517	0.253	0.683	0.633	0.820	0.683	0.746	88	51	108	233
2	2008–2011	2012	0.668	[0.610–0.725]	0.852	0.177	0.189	0.064	0.746	0.771	0.577	0.303	0.450	0.800	0.869	0.450	0.593	84	21	170	139
3	2009–2012	2013	0.619	[0.561–0.681]	0.826	0.182	0.184	0.012	0.757	0.732	0.591	0.290	0.561	0.627	0.824	0.561	0.668	69	41	150	192
4	2010–2013	2014	0.586	[0.523–0.645]	0.769	0.202	0.205	0.014	0.712	0.760	0.521	0.209	0.519	0.627	0.775	0.519	0.622	79	47	150	162
5	2011–2014	2015	0.644	[0.588–0.703]	0.814	0.185	0.195	0.051	0.734	0.767	0.557	0.270	0.476	0.748	0.839	0.476	0.608	92	31	178	162
6	2012–2015	2016	0.633	[0.567–0.699]	0.829	0.173	0.183	0.054	0.760	0.731	0.600	0.313	0.671	0.561	0.829	0.671	0.742	55	43	102	208
7	2013–2016	2017	0.612	[0.554–0.668]	0.814	0.181	0.188	0.037	0.749	0.706	0.582	0.285	0.820	0.395	0.802	0.820	0.811	47	72	64	291
8	2014–2017	2018	0.604	[0.542–0.664]	0.826	0.170	0.174	0.022	0.775	0.669	0.626	0.324	0.869	0.310	0.813	0.869	0.840	35	78	51	339
9	2015–2018	2019	0.667	[0.607–0.720]	0.850	0.170	0.183	0.067	0.760	0.707	0.599	0.326	0.856	0.403	0.819	0.856	0.837	48	71	54	322
10	2016–2019	2020	0.589	[0.531–0.647]	0.809	0.172	0.177	0.025	0.771	0.773	0.618	0.316	0.700	0.465	0.815	0.700	0.753	60	69	130	304
11	2017–2020	2021	0.613	[0.546–0.681]	0.833	0.161	0.167	0.036	0.788	0.749	0.647	0.359	0.845	0.381	0.836	0.845	0.840	37	60	56	305
12	2018–2021	2022	0.595	[0.527–0.660]	0.842	0.159	0.163	0.021	0.796	0.785	0.659	0.366	0.708	0.489	0.844	0.708	0.770	46	48	107	259

Sens, sensitivity; Spec, specificity; Prec, precision; TN, true negative; FP, false positive; FN, false negative; TP, true positive

**Table A4.** Support vector machine results across sliding temporal validation windows

Window	Train	Test	roc_auc	auc_ci	pr_auc	Brier	brier_baseline	brier_skill	Prevalence	thr_youden	nb_max	nb_mean	Sens	Spec	Prec	Recall	F1	TN	FP	FN	TP
1	2007–2010	2011	0.704	[0.653–0.752]	0.830	0.191	0.206	0.073	0.710	0.732	0.517	0.252	0.630	0.712	0.843	0.630	0.721	99	40	126	215
2	2008–2011	2012	0.672	[0.617–0.731]	0.850	0.176	0.189	0.073	0.746	0.713	0.579	0.307	0.689	0.581	0.829	0.689	0.753	61	44	96	213
3	2009–2012	2013	0.605	[0.544–0.665]	0.819	0.181	0.184	0.015	0.757	0.712	0.590	0.287	0.567	0.636	0.829	0.567	0.674	70	40	148	194
4	2010–2013	2014	0.614	[0.556–0.677]	0.766	0.198	0.205	0.032	0.712	0.719	0.518	0.218	0.571	0.683	0.817	0.571	0.672	86	40	134	178
5	2011–2014	2015	0.633	[0.573–0.687]	0.808	0.187	0.195	0.040	0.734	0.746	0.557	0.264	0.524	0.715	0.836	0.524	0.644	88	35	162	178
6	2012–2015	2016	0.589	[0.520–0.656]	0.802	0.179	0.183	0.019	0.760	0.702	0.600	0.291	0.713	0.490	0.815	0.713	0.761	48	50	89	221
7	2013–2016	2017	0.517	[0.456–0.575]	0.750	0.191	0.188	−0.017	0.749	0.745	0.582	0.245	0.468	0.605	0.779	0.468	0.585	72	47	189	166
8	2014–2017	2018	0.613	[0.556–0.671]	0.837	0.170	0.174	0.022	0.775	0.761	0.626	0.324	0.426	0.788	0.874	0.426	0.572	89	24	224	166
9	2015–2018	2019	0.662	[0.608–0.717]	0.841	0.173	0.183	0.050	0.760	0.773	0.599	0.316	0.535	0.756	0.874	0.535	0.663	90	29	175	201
10	2016–2019	2020	0.617	[0.563–0.671]	0.837	0.170	0.177	0.039	0.771	0.793	0.618	0.327	0.438	0.760	0.860	0.438	0.580	98	31	244	190
11	2017–2020	2021	0.593	[0.528–0.654]	0.831	0.166	0.167	0.009	0.788	0.745	0.647	0.344	0.698	0.505	0.840	0.698	0.762	49	48	109	252
12	2018–2021	2022	0.593	[0.525–0.654]	0.840	0.159	0.163	0.021	0.796	0.751	0.659	0.365	0.713	0.457	0.837	0.713	0.770	43	51	105	261

Sens, sensitivity; Spec, specificity; Prec, precision; TN, true negative; FP, false positive; FN, false negative; TP, true positive

**Table A5.** Gradient boosting results across sliding temporal validation windows

Window	Train	Test	roc_auc	auc_ci	pr_auc	Brier	brier_baseline	brier_skill	Prevalence	thr_youden	nb_max	nb_mean	Sens	Spec	Prec	Recall	F1	TN	FP	FN	TP
1	2007–2010	2011	0.677	[0.626–0.726]	0.818	0.194	0.206	0.058	0.710	0.705	0.517	0.242	0.721	0.576	0.807	0.721	0.762	80	59	95	246
2	2008–2011	2012	0.679	[0.627–0.733]	0.862	0.175	0.189	0.075	0.746	0.734	0.577	0.307	0.589	0.695	0.850	0.589	0.696	73	32	127	182
3	2009–2012	2013	0.624	[0.561–0.682]	0.828	0.180	0.184	0.023	0.757	0.737	0.594	0.293	0.541	0.682	0.841	0.541	0.658	75	35	157	185
4	2010–2013	2014	0.590	[0.533–0.645]	0.777	0.199	0.205	0.029	0.712	0.658	0.521	0.219	0.843	0.310	0.751	0.843	0.795	39	87	49	263
5	2011–2014	2015	0.622	[0.554–0.673]	0.794	0.186	0.195	0.045	0.734	0.707	0.557	0.264	0.771	0.423	0.787	0.771	0.779	52	71	78	262
6	2012–2015	2016	0.646	[0.581–0.709]	0.822	0.174	0.183	0.048	0.760	0.718	0.600	0.311	0.752	0.490	0.823	0.752	0.786	48	50	77	233
7	2013–2016	2017	0.614	[0.553–0.671]	0.820	0.181	0.188	0.036	0.749	0.759	0.582	0.284	0.541	0.639	0.817	0.541	0.651	76	43	163	192
8	2014–2017	2018	0.597	[0.540–0.659]	0.821	0.172	0.174	0.015	0.775	0.702	0.626	0.319	0.805	0.381	0.818	0.805	0.811	43	70	76	314
9	2015–2018	2019	0.650	[0.592–0.697]	0.847	0.175	0.183	0.041	0.760	0.778	0.599	0.310	0.489	0.756	0.864	0.489	0.625	90	29	192	184
10	2016–2019	2020	0.585	[0.520–0.645]	0.809	0.172	0.177	0.026	0.771	0.757	0.618	0.317	0.783	0.403	0.815	0.783	0.799	52	77	94	340
11	2017–2020	2021	0.613	[0.547–0.674]	0.833	0.163	0.167	0.026	0.788	0.766	0.647	0.353	0.676	0.515	0.838	0.676	0.748	50	47	117	244
12	2018–2021	2022	0.579	[0.514–0.643]	0.831	0.159	0.163	0.023	0.796	0.787	0.659	0.365	0.612	0.553	0.842	0.612	0.709	52	42	142	224

Sens, sensitivity; Spec, specificity; Prec, precision; TN, true negative; FP, false positive; FN, false negative; TP, true positive

**Table A6.** Majority-class (survival) performance metrics across sliding temporal validation windows. Metrics are computed considering survival (class 1) as the positive class.

Window	Train	Test	roc_auc	auc_ci	pr_auc	Brier	brier_baseline	brier_skill	Prevalence	thr_youden	nb_max	nb_mean	Sens	Spec	Prec	Recall	F1	TN	F	FN	TP
1	2007–2010	2011	0.618	[0.563–0.676]	0.778	0.216	0.206	−0.052	0.710	0.610	0.487	0.206	0.683	0.518	0.777	0.683	0.727	72	67	108	233
2	2008–2011	2012	0.631	[0.574–0.693]	0.814	0.227	0.189	−0.199	0.746	0.396	0.527	0.183	0.867	0.352	0.798	0.867	0.831	37	68	41	268
3	2009–2012	2013	0.585	[0.520–0.650]	0.801	0.227	0.184	−0.231	0.757	0.568	0.534	0.193	0.693	0.473	0.803	0.693	0.744	52	58	105	237
4	2010–2013	2014	0.572	[0.511–0.634]	0.742	0.234	0.205	−0.140	0.712	0.571	0.470	0.144	0.718	0.437	0.759	0.718	0.738	55	71	88	224
5	2011–2014	2015	0.567	[0.506–0.627]	0.755	0.221	0.195	−0.133	0.734	0.568	0.528	0.175	0.768	0.382	0.774	0.768	0.771	47	76	79	261
6	2012–2015	2016	0.580	[0.508–0.656]	0.802	0.235	0.183	−0.290	0.760	0.513	0.565	0.171	0.794	0.439	0.817	0.794	0.805	43	55	64	246
7	2013–2016	2017	0.574	[0.500–0.631]	0.778	0.200	0.188	−0.062	0.749	0.633	0.567	0.228	0.817	0.345	0.788	0.817	0.802	41	78	65	290
8	2014–2017	2018	0.609	[0.549–0.669]	0.827	0.202	0.174	−0.162	0.775	0.562	0.592	0.248	0.815	0.372	0.817	0.815	0.816	42	71	72	318
9	2015–2018	2019	0.630	[0.570–0.690]	0.821	0.208	0.183	−0.138	0.760	0.571	0.580	0.234	0.803	0.445	0.821	0.803	0.812	53	66	74	302
10	2016–2019	2020	0.577	[0.519–0.633]	0.806	0.201	0.177	−0.136	0.771	0.702	0.594	0.251	0.433	0.705	0.832	0.433	0.570	91	38	246	188
11	2017–2020	2021	0.570	[0.502–0.636]	0.815	0.211	0.167	−0.262	0.788	0.553	0.632	0.224	0.861	0.351	0.832	0.861	0.846	34	63	50	311
12	2018–2021	2022	0.558	[0.489–0.613]	0.823	0.204	0.163	−0.254	0.796	0.537	0.642	0.242	0.863	0.255	0.819	0.863	0.840	24	70	50	316

Sens, sensitivity; Spec, specificity; Prec, precision; TN, true negative; FP, false positive; FN, false negative; TP, true positive

**Table A7.** Minority-class (mortality) performance metrics across sliding temporal validation windows. Metrics are computed considering death (class 0) as the positive class.

Window	Train	Test	roc_auc	auc_ci	pr_auc	Brier	brier_base	brier_skill	Prev	thr	nb_max	nb_mean	Sens	Spec	Prec	Rec	F1	TN	FP	FN	TP
1	2007–2010	2011	0.382	[0.324–0.437]	0.239	0.371	0.206	−0.804	0.290	0.122	−0.176	−0.411	0.993	0.012	0.291	0.993	0.450	4	337	1	138
2	2008–2011	2012	0.369	[0.307–0.426]	0.203	0.321	0.189	−0.696	0.254	inf	−0.021	−0.233	0.000	1.000	0.000	0.000	0.000	309	0	105	0
3	2009–2012	2013	0.415	[0.350–0.480]	0.210	0.332	0.184	−0.804	0.243	0.045	−0.084	−0.300	1.000	0.006	0.244	1.000	0.393	2	340	0	110
4	2010–2013	2014	0.428	[0.366–0.489]	0.274	0.307	0.205	−0.500	0.288	0.816	−0.012	−0.189	0.032	0.990	0.571	0.032	0.060	309	3	122	4
5	2011–2014	2015	0.433	[0.373–0.494]	0.257	0.336	0.195	−0.721	0.266	0.773	−0.029	−0.290	0.122	0.918	0.349	0.122	0.181	312	28	108	15
6	2012–2015	2016	0.420	[0.344–0.492]	0.215	0.321	0.183	−0.756	0.240	0.564	−0.051	−0.235	0.357	0.700	0.273	0.357	0.310	217	93	63	35
7	2013–2016	2017	0.426	[0.369–0.500]	0.235	0.371	0.188	−0.974	0.251	0.898	−0.011	−0.409	0.017	0.997	0.667	0.017	0.033	354	1	117	2
8	2014–2017	2018	0.391	[0.331–0.451]	0.185	0.359	0.174	−1.063	0.225	inf	−0.080	−0.384	0.000	1.000	0.000	0.000	0.000	390	0	113	0
9	2015–2018	2019	0.370	[0.310–0.430]	0.195	0.360	0.183	−0.970	0.240	0.119	−0.088	−0.386	1.000	0.005	0.241	1.000	0.389	2	374	0	119
10	2016–2019	2020	0.423	[0.367–0.481]	0.203	0.375	0.177	−1.123	0.229	0.872	−0.059	−0.439	0.039	0.975	0.313	0.039	0.069	423	11	124	5
11	2017–2020	2021	0.430	[0.364–0.498]	0.197	0.339	0.167	−1.030	0.212	0.596	−0.033	−0.322	0.340	0.687	0.226	0.340	0.272	248	113	64	33
12	2018–2021	2022	0.442	[0.387–0.511]	0.184	0.349	0.163	−1.143	0.204	inf	−0.063	−0.352	0.000	1.000	0.000	0.000	0.000	366	0	94	0

Prev, prevalence; Sens, sensitivity; Spec, specificity; Prec, precision; TN, true negative; FP, false positive; FN, false negative; TP, true positive

## Appendix B. Baseline characteristics according to survival outcome

**Table B1.** Baseline characteristics stratified by 12-month survival outcome. Continuous variables are presented as median (IQR), and categorical variables, as *n* (%). Most nonsignificant variables were omitted for brevity.

Variable	Overall	Survival ≥12 months	Death <12 months	*P* value
Current updated MELD score	29.00 (24.00–31.00)	29.00 (24.00–29.00)	29.00 (24.00–35.00)	<0.001
Prioritization	6,963 (91.3%)	5,218 (93.3%)	1,745 (85.5%)	<0.001
Diagnosis (HBV/HCV cirrhosis)	2,524 (33.1%)	1,892 (33.8%)	632 (31.0%)	<0.001
Recipient height (cm)	169.00 (163.00–175.00)	169.00 (163.00–175.00)	168.00 (160.00–174.00)	<0.001
Donor ABO (O)	3,742 (49.0%)	2,627 (47.0%)	1,115 (54.7%)	<0.001
Male recipient	5,270 (69.1%)	3,953 (70.7%)	1,317 (64.6%)	<0.001
Injection drug use (recipient)	6,752 (88.5%)	4,997 (89.4%)	1,755 (86.0%)	<0.001
Anti-HBc (record)	0.00 (0.00–0.00)	0.00 (0.00–0.00)	0.00 (0.00–0.00)	<0.001
Toxoplasmosis IgM	1.00 (1.00–1.00)	1.00 (1.00–1.00)	1.00 (1.00–1.00)	<0.001
Recipient age at transplant	55.00 (46.00–62.00)	55.00 (46.00–61.00)	56.00 (46.00–63.00)	<0.001
Recipient weight (kg)	75.00 (65.00–86.00)	75.00 (65.00–86.00)	74.00 (64.00–85.00)	0.002
Recipient ABO (O)	3,295 (43.2%)	2,350 (42.0%)	945 (46.3%)	0.002
Anti-HBs negative (recipient)	6,823 (89.4%)	4,963 (88.8%)	1,860 (91.2%)	0.002
Donor AST (TGO)	52.00 (31.00–95.00)	53.00 (31.00–97.00)	49.00 (30.00–88.00)	0.003
White recipient	5,637 (73.9%)	4,101 (73.4%)	1,536 (75.3%)	0.012
Donor FiO_2_	100.00 (100.00–100.00)	100.00 (100.00–100.00)	100.00 (100.00–100.00)	0.012
Local donor infection	0.00 (0.00–1.00)	0.00 (0.00–1.00)	0.00 (0.00–1.00)	0.013
No chronic alcohol consumption	6,501 (85.2%)	4,729 (84.6%)	1,772 (86.9%)	0.013
VDRL	1.00 (1.00–1.00)	1.00 (1.00–1.00)	1.00 (1.00–1.00)	0.021
CMV IgM	1.00 (1.00–1.00)	1.00 (1.00–1.00)	1.00 (1.00–1.00)	0.025
Donor anti-HBc (not evaluated)	7,202 (94.4%)	5,258 (94.1%)	1,944 (95.3%)	0.030
Cause of death (stroke)	4,159 (54.5%)	3,007 (53.8%)	1,152 (56.5%)	0.034
Donor amylase	81.00 (45.00–168.00)	81.00 (45.00–173.00)	80.05 (45.00–154.00)	0.042
Anti-HCV negative (recipient)	6,215 (81.5%)	4,523 (80.9%)	1,692 (82.9%)	0.046
Donor weight (kg)	71.00 (65.00–80.00)	72.00 (65.00–80.00)	70.00 (65.00–80.00)	0.065
Organ retrieval time (07:00:00)	184 (2.4%)	128 (2.3%)	56 (2.7%)	0.089
Total ischemia time (07:00:00)	184 (2.4%)	128 (2.3%)	56 (2.7%)	0.089
Donor height (cm)	170.00 (162.00–175.00)	170.00 (162.00–175.00)	168.00 (160.00–175.00)	0.125
Hematocrit (recipient)	33.20 (27.80–38.90)	33.00 (27.60–38.90)	33.40 (28.00–39.00)	0.134
Creatine phosphokinase (donor)	594.00 (209.25–1,612.75)	599.00 (213.00–1,646.50)	577.00 (198.00–1,541.00)	0.164
Donor age (years)	43.00 (29.00–54.00)	43.00 (29.00–53.00)	44.00 (30.00–54.00)	0.184
Creatinine (donor)	1.30 (0.88–2.12)	1.30 (0.88–2.18)	1.30 (0.86–2.07)	0.196
Intubation duration (d)	4.00 (3.00–6.00)	4.00 (3.00–6.75)	4.00 (3.00–6.00)	0.207
Total bilirubin (donor)	0.47 (0.30–0.72)	0.47 (0.30–0.72)	0.48 (0.30–0.74)	0.540
Diabetes	0.00 (0.00–0.00)	0.00 (0.00–0.00)	0.00 (0.00–0.00)	0.671
Noradrenaline use (donor)	1.00 (1.00–1.00)	1.00 (1.00–1.00)	1.00 (1.00–1.00)	0.674
ICU length of stay (d)	4.00 (3.00–7.00)	4.00 (3.00–7.00)	4.00 (3.00–7.00)	0.688
Direct bilirubin (donor)	0.20 (0.11–0.36)	0.20 (0.11–0.36)	0.20 (0.11–0.36)	0.688
Cold ischemia time (recipient)	12.00 (8.00–12.00)	12.00 (8.00–12.00)	12.00 (8.00–12.00)	0.870

IQR, interquartile range; MELD, Model for End-Stage Liver Disease; HBV, hepatitis B virus; HCV, hepatitis C virus; anti-HBc, antibody to hepatitis B core antigen; IgM, immunoglobulin M; anti-HBs, antibody to hepatitis B surface antigen; AST, aspartate aminotransferase; TGO, glutamic-oxaloacetic transaminase; VDRL, Venereal Disease Research Laboratory; CMV, cytomegalovirus; ICU, intensive care unit

## Appendix C. Hyperparameter search spaces and hyperparameters by window

**Table C1.** Hyperparameters search space by model

Model	Hyperparameters
Random forest	class_weight = “balanced_subsample”
	n_estimators: randint(600, 1200)
	max_depth: randint(3, 10)
	min_samples_leaf: randint(15, 60)
	min_samples_split: randint(20, 80)
	max_features: [“sqrt”, 0.3, 0.5]
	max_samples: [0.6, 0.7, 0.8, None]
	n_iter = 40
	scoring = “average_precision”
XGBoost	objective = “binary:logistic”
	eval_metric = “aucpr”
	tree_method = “hist”
	scale_pos_weight = $n_{\mathrm{neg}}/\left(n_{\mathrm{pos}}+10^{-9}\right)$
	learning_rate: loguniform(0.01, 0.08)
	max_depth: randint(3, 5)
	min_child_weight: randint(15, 50)
	subsample: [0.6, 0.8]
	colsample_bytree: [0.6, 0.8]
	gamma: loguniform(0.01, 1.0)
	reg_alpha: loguniform(0.1, 5)
	reg_lambda: loguniform(2, 15)
	n_iter = 40
	scoring = “average_precision”
SVM	class_weight = “balanced”
	kernel = rbf
	svm__C: loguniform(1e−2, 100)
	svm__gamma: loguniform(1e−4, 5)
	kernel = linear
	svm__C: loguniform(1e−3, 50)
	kernel = poly
	svm__C: loguniform(1e−2, 50)
	svm__gamma: loguniform(1e−4, 1)
	svm__degree: [2–4]
	svm__coef0: [0, 0.5, 1]
	n_iter = 40
	scoring = “average_precision”
Logistic regression	class_weight = “balanced”
	solver = “saga”
	penalty = “elasticnet”
	max_iter = 5,000
	logreg__C: loguniform(1e−2, 3)
	logreg__l1_ratio: [0.0, 0.2, 0.5, 0.8, 1.0]
	n_iter = 40
	scoring = “average_precision”
Histogram gradient boosting	class_weight=“balanced”
	validation_fraction = 0.1
	n_iter_no_change = 20
	scoring = “average_precision”
	learning_rate: loguniform(0.01, 0.08)
	max_iter: randint(800, 2000)
	max_depth: randint(3, 6)
	max_leaf_nodes: randint(20, 80)
	min_samples_leaf: randint(15, 50)
	l2_regularization: loguniform(0.1, 5)

SVM, support vector machine

**Table C2.** HGBM hyperparameters by temporal window

Window	Train	Test	L2 reg.	LR	Depth	Iter	Leaf nodes	Min leaf
1	2007–2010	2011	0.1479	0.0260	3	1,923	59	38
2	2008–2011	2012	0.4290	0.0706	3	1,713	65	32
3	2009–2012	2013	0.8987	0.0497	5	1,594	28	29
4	2010–2013	2014	1.1197	0.0357	3	1,547	44	41
5	2011–2014	2015	0.4328	0.0722	5	1,895	40	33
6	2012–2015	2016	0.5326	0.0105	3	1,663	58	46
7	2013–2016	2017	4.9273	0.0744	3	1,860	51	23
8	2014–2017	2018	0.4290	0.0706	3	1,713	65	32
9	2015–2018	2019	1.5194	0.0134	5	1,548	34	43
10	2016–2019	2020	1.2325	0.0119	4	903	39	49
11	2017–2020	2021	0.4290	0.0706	3	1,713	65	32
12	2018–2021	2022	0.5326	0.0105	3	1,663	58	46

LR, logistic regression; HGBM, histogram-based gradient boosting machine

**Table C3.** Logistic regression hyperparameters by temporal window

Window	Train	Test	*C*	L1 ratio
1	2007–2010	2011	0.2548	1.0
2	2008–2011	2012	0.1373	1.0
3	2009–2012	2013	0.2548	1.0
4	2010–2013	2014	0.8184	0.8
5	2011–2014	2015	0.3083	0.5
6	2012–2015	2016	1.3495	1.0
7	2013–2016	2017	0.1373	1.0
8	2014–2017	2018	0.1231	0.8
9	2015–2018	2019	0.2548	1.0
10	2016–2019	2020	0.3083	0.5
11	2017–2020	2021	0.0153	0.0
12	2018–2021	2022	0.0847	1.0

**Table C4.** SVM hyperparameters by temporal window

Window	Train	Test	*C*	Kernel
1	2007–2010	2011	0.0719	Linear
2	2008–2011	2012	0.0719	Linear
3	2009–2012	2013	0.3707	Linear
4	2010–2013	2014	0.0719	Linear
5	2011–2014	2015	0.0995	Linear
6	2012–2015	2016	0.0099	Linear
7	2013–2016	2017	0.0099	Linear
8	2014–2017	2018	4.3886	Linear
9	2015–2018	2019	0.0719	Linear
10	2016–2019	2020	0.0719	Linear
11	2017–2020	2021	0.0087	Linear
12	2018–2021	2022	0.0045	Linear

SVM, support vector machine

**Table C5.** XGBoost hyperparameters by temporal window

Window	Train	Test	Colsample	Gamma	LR	Depth	Min child	Alpha	Lambda	Subsample
1	2007–2010	2011	0.6	0.0152	0.0568	4	20	1.5211	11.2758	0.8
2	2008–2011	2012	0.6	0.2766	0.0190	4	26	0.7319	7.2088	0.8
3	2009–2012	2013	0.6	0.0624	0.0293	4	15	1.4060	8.7981	0.8
4	2010–2013	2014	0.8	0.1513	0.0766	3	22	1.1811	9.9206	0.8
5	2011–2014	2015	0.6	0.0583	0.0754	3	15	1.6834	3.2176	0.8
6	2012–2015	2016	0.6	0.6197	0.0194	3	15	0.2439	4.7291	0.8
7	2013–2016	2017	0.8	0.1513	0.0766	3	22	1.1811	9.9206	0.8
8	2014–2017	2018	0.6	0.0583	0.0754	3	15	1.6834	3.2176	0.8
9	2015–2018	2019	0.8	0.1513	0.0766	3	22	1.1811	9.9206	0.8
10	2016–2019	2020	0.8	0.1513	0.0766	3	22	1.1811	9.9206	0.8
11	2017–2020	2021	0.6	0.5399	0.0349	4	17	0.1084	14.1176	0.8
12	2018–2021	2022	0.6	0.0624	0.0293	4	15	1.4060	8.7981	0.8

LR, logistic regression

**Table C6.** Random forest hyperparameters by temporal window

Window	Train	Test	Depth	Max features	Max samples	Min leaf	Min split	Estimators
1	2007–2010	2011	4	0.3	1.0	37	56	824
2	2008–2011	2012	3	sqrt	0.7	26	58	985
3	2009–2012	2013	6	0.3	1.0	18	21	989
4	2010–2013	2014	4	sqrt	1.0	28	69	945
5	2011–2014	2015	6	sqrt	1.0	18	49	1,084
6	2012–2015	2016	3	sqrt	0.7	26	58	985
7	2013–2016	2017	3	sqrt	0.7	26	58	985
8	2014–2017	2018	5	sqrt	0.6	31	78	712
9	2015–2018	2019	7	sqrt	0.8	56	63	973
10	2016–2019	2020	6	sqrt	0.6	43	34	664
11	2017–2020	2021	3	sqrt	0.7	26	58	985
12	2018–2021	2022	3	sqrt	0.7	26	58	985

## Appendix D. Interpretation of UMAP hyperparameters for the transplant dataset

**Table:**

Parameter	Interpretation
metric: manhattan	Medical datasets often exhibit asymmetric distributions and physiological outliers. The L1 geometry captures patient similarity more effectively. The Manhattan metric is also more robust to outliers.
n_neighbors: 15	Using 15 neighbors enables the identification of clinically meaningful subgroups. This represents an appropriate clinical scale for defining proximity.
min_dist: 0.0	Patients within the same class (e.g., survival ≥12 months vs. <12 months) become extremely close in the embedding, indicating a real concentration of similar clinical patterns.
target_weight: 0.0	UMAP was applied in a fully unsupervised manner, relying exclusively on the intrinsic structure of the input features without incorporating class label information.
spread: 0.5	The resulting clusters are compact and well-defined, with minimal distortion.
local_conn: 10	Each patient maintains at least 10 reliable local connections, preventing graph discontinuities. This reflects a strong and continuous local structure in the dataset.
init: spectral	Spectral initialization provides a stable, reliable, and consistent embedding across multiple runs.
densmap: False	For clinical datasets, class separation is more important than preserving true density. This setting is well suited for visualization and clinical interpretation.
